# Hepatoviruses promote very-long-chain fatty acid and sphingolipid synthesis for viral RNA replication and quasi-enveloped virus release

**DOI:** 10.1126/sciadv.adj4198

**Published:** 2023-10-20

**Authors:** Tomoyuki Shiota, Zhucui Li, Guan-Yuan Chen, Kevin L. McKnight, Takayoshi Shirasaki, Bryan Yonish, Heyjeong Kim, Ethan J. Fritch, Timothy P. Sheahan, Masamichi Muramatsu, Maryna Kapustina, Craig E. Cameron, You Li, Qibin Zhang, Stanley M. Lemon

**Affiliations:** ^1^ Lineberger Comprehensive Cancer Center, The University of North Carolina at Chapel Hill, Chapel Hill, NC, USA.; ^2^ Center for Translational Biomedical Research, The University of North Carolina at Greensboro, Kannapolis, NC, USA.; ^3^ Department of Microbiology and Immunology, The University of North Carolina at Chapel Hill, Chapel Hill, NC, USA.; ^4^ Department of Epidemiology, The University of North Carolina at Chapel Hill, Chapel Hill, NC, USA.; ^5^ Department of Infectious Disease Research, Foundation for Biomedical Research and Innovation at Kobe, Kobe, Hyogo, Japan.; ^6^ Department of Cell Biology and Physiology, The University of North Carolina at Chapel Hill, Chapel Hill, NC, USA.; ^7^ Department of Medicine, The University of North Carolina at Chapel Hill, Chapel Hill, NC, USA.; ^8^ Department of Chemistry and Biochemistry, The University of North Carolina at Greensboro, Greensboro, NC, USA.

## Abstract

Virus-induced changes in host lipid metabolism are an important but poorly understood aspect of viral pathogenesis. By combining nontargeted lipidomics analyses of infected cells and purified extracellular quasi-enveloped virions with high-throughput RNA sequencing and genetic depletion studies, we show that hepatitis A virus, an hepatotropic picornavirus, broadly manipulates the host cell lipid environment, enhancing synthesis of ceramides and other sphingolipids and transcriptionally activating acyl–coenzyme A synthetases and fatty acid elongases to import and activate long-chain fatty acids for entry into the fatty acid elongation cycle. Phospholipids with very-long-chain acyl tails (>C22) are essential for genome replication, whereas increases in sphingolipids support assembly and release of quasi-enveloped virions wrapped in membranes highly enriched for sphingomyelin and very-long-chain ceramides. Our data provide insight into how a pathogenic virus alters lipid flux in infected hepatocytes and demonstrate a distinction between lipid species required for viral RNA synthesis versus nonlytic quasi-enveloped virus release.

## Teaser

High-resolution LC/MS reveals increased >C22 fatty acids and quasi-enveloped virions enriched in ceramides and sphingomyelin.

## INTRODUCTION

Despite excellent vaccines, hepatitis A virus (HAV) remains a common cause of enterically transmitted viral hepatitis and a global threat to public health (*1*, *2*). Classified in the genus *Hepatovirus*, the virus is an atypical hepatotropic member of the *Picornaviridae*, distinct from enteroviruses such as poliovirus in its phylogeny, structure, and replication strategy (*3*, *4*). HAV replicates efficiently in hepatocytes without cytopathic effect, with liver injury resulting eventually from innate and adaptive immune responses. As with other positive-strand RNA viruses, new copies of the RNA genome are synthesized in association with a cytoplasmic membranous network formed from the endoplasmic reticulum or Golgi (*5*, *6*). Following packaging of the genome, newly assembled capsids bud into endosomes that subsequently fuse with the plasma membrane, releasing capsids into the bloodstream and biliary system enclosed in small vesicles resembling exosomes (*7*, *8*). High concentrations of bile salts disrupt vesicles released across the apical hepatocyte membrane into the biliary system, leading to fecal shedding of canonical “naked” capsids, but stable membrane-wrapped “quasi-enveloped” virions (eHAV) are the only particle type found circulating in the blood during acute hepatitis A (*7*, *9*). These vesicle-like virions lack virus-encoded proteins on their surface (*10*), yet they are infectious and account for further spread of the virus within the liver.

The host cell membranes of hepatocytes thus figure prominently in the life cycle of hepatoviruses, providing both sites of genome replication and a mechanism for nonlytic viral egress. Despite this, nothing is known about whether and how HAV manipulates the complex lipid metabolism of the hepatocyte to promote its replication, nor what lipid species comprise the membranes of the quasi-enveloped virion. This is a particularly important question, as increasing numbers of other canonical “nonenveloped” viruses have been shown to be released from cells enclosed within extracellular vesicles with substantial implications for pathogenesis and host immune responses (*11*–*14*). Recent advances in high-resolution mass spectrometry–based lipidomics offer the potential to gain a greater understanding of the role played by lipid metabolism in viral pathogenesis (*15*, *16*). Here, we describe global lipidomics analyses of infected human hepatoma cells and purified extracellular eHAV virions, coupled with transcriptomic analyses and genetic depletion studies to elucidate how HAV modifies hepatocellular lipid metabolism to facilitate genome replication and quasi-enveloped virus release. We show that infection induces a transcriptional program that promotes the synthesis of phospholipids with very-long-chain acyl tails (>C22) essential for replication of the viral RNA genome and enhances the abundance of sphingolipids including very-long-chain ceramides (Cer) that are highly enriched in extracellular quasi-enveloped virions.

## RESULTS

### Global lipidomic changes induced by HAV infection

**Fig. 1.**
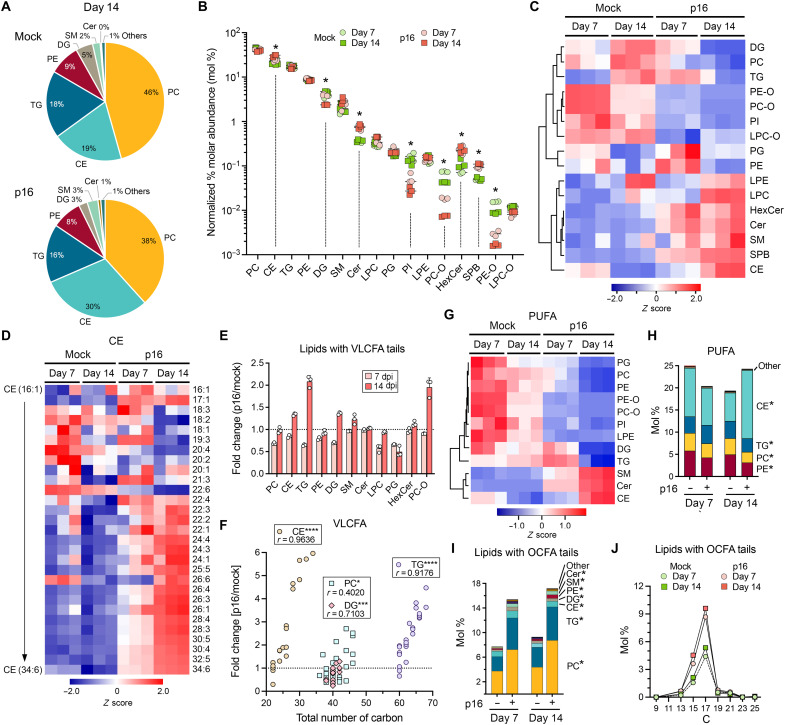
Quantitative lipidomics analysis of HAV p16-infected hepatoma cells. (**A**) Relative molar abundance (mol %) of major lipid classes 14 days in (top) mock-infected or (bottom) p16 virus–infected Huh-7.5 cells. SM, sphingomyelin. (**B**) Major lipid classes in triplicate cell samples collected 7 and 14 days after mock or p16 virus infection, rank ordered by relative abundance in uninfected cells on day 7: LPC, lysophosphatidylcholine; PG, phosphatidylglycerol; PI, phosphatidylinositol; LPE, lysophosphatidylethanolamine; PC-O, ether-linked phosphatidylcholine; SPB, sphingosine; PE-O, ether-linked phosphatidylethanolamine; LPC-O, ether-linked lysophosphatidylcholine. *50% increase or decrease from mock at day 14, with *Q* < 0.01. (**C**) Heatmap of major lipid classes, with clustering based on Spearman rank order correlation (*65*). (**D**) Heatmap showing molar abundance of different CE species grouped by ascending total carbon number. (**E**) Fold change in percent molar abundance of lipids with very-long-chain fatty acid (VLCFA) tails >22 carbons within each lipid class at 7 and 14 dpi. Data shown are means ± SD. (**F**) Fold change in molar abundance of CE, PC, DG, and TG with VLCFA tails 14 dpi plotted as a function of the total number of carbon atoms in acyl tails. *P < 0.05, ***P < 0.001, ****P < 0.0001 by Spearman correlation. (**G**) Heatmap of lipid species with >3 double bonds (PUFA) with lipid class grouped by Spearman rank correlation. (**H**) Relative molar abundance of PUFA by lipid class. *Q < 0.01 at both 7 and 14 dpi. (**I**) Relative molar abundance of lipids with odd-number carbon fatty acid (OCFA) tails in mock- and p16-infected cells at 7 and 14 dpi. *Q* < 0.01 at both 7 and 14 dpi. (**J**) Percent molar abundance of lipids containing OCFA tails with defined numbers of carbon atoms.

To assess the impact of HAV infection on host cell lipid metabolism, we characterized the lipid composition of Huh-7.5 hepatoma cells 7 and 14 days postinfection (dpi) with HM175/p16 virus (hereafter “p16”), a virus with only limited passage in cell culture (*17*). Confocal microscopy demonstrated the presence of HAV antigen in most, if not all, cells at both time points (fig. S1A). Triplicate cell harvests were extracted for lipids and subjected to untargeted high-resolution liquid chromatography–tandem mass spectrometry (LC-MS/MS) together with samples from mock-infected cells cultured in parallel (see Materials and Methods). A total of 688 individual lipid species were identified in 16 lipid classes, with principal components analysis (PCA) of normalized data demonstrating clear clustering of samples along the major axis by time and infection (fig. S1, B and C, and table S1). Phosphatidylcholine (PC) was the most abundant lipid in both infected and uninfected cells, followed by cholesterol esters (CEs), triglycerides (TGs), phosphatidylethanolamine (PE), and diglycerides (DGs) (Fig. 1, A and B). By 14 dpi, the relative abundance of 8 of 16 lipid classes was altered more than 50% (*Q* < 0.01%), with lesser changes evident at 7 dpi (Fig. 1, B and C). Among more abundant lipids, CEs were increased and DGs decreased. While of relatively low overall abundance, Cer, hexosylceramides, and sphingosines (SPBs) were each increased greater than twofold, whereas phosphatidylinositol (PI) and ether-linked PC (PC-O) and PE (PE-O) were equivalently decreased (Fig. 1, B and C).

Infection caused a notable increase in phospholipids with very-long-chain fatty acid (VLCFA) tails containing >22 carbons. On a molar basis, such lipids represented only 13.6 ± 1.1 (SD) percent (mol %) of those identified in mock-infected cells but accounted for 22.3 ± 1.7 mol % by 14 dpi (*P* = 0.003). Lipids with VLCFA tails were increased in multiple classes, including in particular CE, DG, TG, and PC-O (Fig. 1, D and E, and fig. S2, A and B), with the degree of enrichment correlating positively with chain length (Fig. 1F). Other notable changes included a decrease in phospholipids with polyunsaturated acyl tails containing more than three double bonds (PUFA), coupled with an increase in PUFA among sphingomyelin (SM), Cer, and CE (Fig. 1, G and H, and fig. S2, A to F). Lipids with acyl tails containing odd numbers of carbons, generally rare in mammalian cells, were also broadly increased (Fig. 1, I and J, and fig. S2, A to D and F). For the most part, this reflected infection-driven increases in C17 and C15 odd-number carbon fatty acids (OCFAs) (Fig. 1J). Collectively, these results show a rewiring of lipid metabolism leading to a greater abundance of sphingolipids and phospholipids containing VLCFA tails, and altered proportions of PUFA and OCFA.

### VLCFA and HAV genome replication

**Fig. 2.**
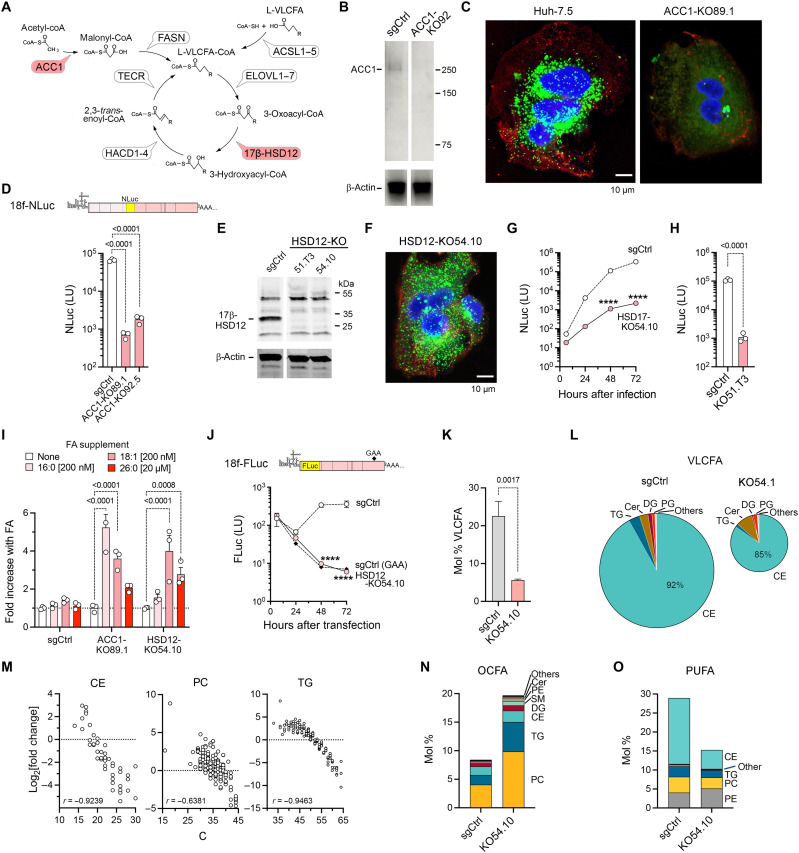
VLCFA synthesis is required for hepatovirus replication. (**A**) VLCFA synthesis pathway. (**B**) Immunoblots of ACC1 in lysates of ACC1-KO92 and control (sgCtrl) cells. (**C**) Maximum intensity projection confocal images of clonal ACC1-KO89.1 cells with targeted CRISPR deletion of *ACACA* and parental Huh-7.5 cells showing lipid droplets stained with BODIPY (green) and CellMask membrane dye (red). HexCer, hexosylceramide. (**D**) Top: 18f-NLuc HAV reporter virus genome. Bottom: NLuc expressed by 18f-NLuc virus in clonal ACC1-depleted cell lines, ACC1-KO89.1 and ACC1-KO92.5, 72 hours after infection. (**E**) Immunoblot showing 17β-HSD12 expression in cells with targeted CRISPR deletion of *HSD17B12* (encoding 17β-HSD12) and sgCtrl cells. (**F**) Maximum intensity projection confocal image of HSD12-KO54.10 cells stained with BODIPY-C9. (**G** and **H**) NLuc expression in 18f-NLuc–infected HSD17-KO54.10 (G) or HSD17-KO51.T3 (H) versus control sgCtrl cells. ****P < 0.0001. (**I**) Fold increase in NLuc expressed by 18f-NLuc virus 72 hours after infection of ACC1-depleted, 17β-HSD12–depleted, or sgCtrl cells supplemented with fatty acids. (**J**) Top: Subgenomic 18f-FLuc replicon RNA; GAA mutation ablates replication. Bottom: FLuc expressed by HSD12-KO54.10 and sgCtrl cells transfected with 18f-FLuc RNA or sgCtrl cells transfected with 18f-FLuc/GAA. ****P < 0.0001. (**K**) Relative molar abundance of lipids with VLCFA tails in HSD12-KO54.10 and sgCtrl cells. (**L**) Class distribution of lipids with VLCFA tails in sgCtrl and 17β-HSD12–depleted cells. Pie chart area is proportional to molar abundance. (**M**) Fold change in relative molar abundance of CE, PC, and TG with VLCFA tails plotted against numbers of carbon atoms in acyl tails. s = Spearman correlation (*P* < 0.0001 for each class). (**N**) Relative molar abundance of lipids with odd-number carbon fatty acid (OCFA) tails in sgCtrl and 17β-HSD12–depleted cells. *Q* < 0.05 for each lipid class shown. (**O**) Relative molar abundance of polyunsaturated lipids (>3 double bonds) in sgCtrl and 17β-HSD12–depleted cells. *Q* < 0.05 for each class shown.

Clues to lipids specifically required for hepatovirus replication can be found in a recent genome-wide CRISPR screen that identified, among other HAV host factors, acetyl–coenzyme A (CoA) carboxylase 1 (ACC1, encoded by *ACACA*) and very-long-chain 3-oxoacyl-CoA reductase, otherwise known as 17β-hydroxysteroid dehydrogenase 12 (17β-HSD12, encoded by *HSD17B12*) (*18*) (fig. S3, A and B). ACC1 catalyzes the carboxylation of acetyl-CoA to malonyl-CoA, the first and rate-limiting step in de novo fatty acid synthesis, while 17β-HSD12 is an essential component of the fatty acid elongation cycle, catalyzing the second of four reactions that add two carbons to long-chain fatty acids (>14 carbon atoms) and VLCFA in each cycle (*19*, *20*) (Fig. 2A). Targeted deletion of either gene significantly impaired HAV replication. Clonally isolated Huh-7.5 cells with CRISPR depletion of ACC1 (ACC1-KO89.1 and ACC1-92.5) proliferated poorly and contained few lipid droplets unless supplemented with long-chain fatty acids: palmitic acid (hexadecanoic acid, 16:0) or oleic acid (cis-9-octadecenoic acid, 18:1) (Fig. 2, B and C). These cells supported replication of a reporter virus (18f-NLuc) (*21*) at levels ~100-fold less than ACC1-replete control cells transduced with nontargeting single-guide RNA (sgCtrl cells) (Fig. 2D). By contrast, type 1 poliovirus (PV1, like HAV a picornavirus), replicated well in the ACC1-depleted cells, indicating that the defect in HAV replication was not related to the health of the cells (fig. S3C). Consistent with these data, HAV replication was potently inhibited by the ACC1 inhibitor, firsocostat (fig. S3D).

Unlike the ACC1-depleted cells, cells with targeted 17β-HSD12 depletion (HSD12-KO54.10 and HSD12-K051.T3) proliferated well without fatty acid supplements and contained numerous lipid droplets only slightly smaller than those in sgCtrl cells (Fig. 2, E and F, and fig. S4, A and B). Nonetheless, these cells were equally nonpermissive for HAV infection (Fig. 2, G and H). Reporter virus replication was enhanced in both ACC1- and 17β-HSD12B–depleted cells by supplementation with oleic acid, palmitic acid, or the VLCFA, hexacosanoic acid (26:0), whereas similar supplementation of sgCtrl cells resulted in only minimal increases (Fig. 2I). Collectively, these data show that hepatoviruses are highly dependent on both ACC1 and 17β-HSD12 for infection.

A subgenomic HAV replicon RNA (18f-FLuc) (*22*) was fully competent translationally but nonetheless failed to replicate when transfected into 17β-HSD12–depleted cells (Fig. 2J), indicating a defect in RNA synthesis. By contrast, PV1 and a similar subgenomic PV1 replicon replicated well in 17β-HSD12–depleted Huh-7.5 cells (fig. S3C), as did severe acute respiratory syndrome coronavirus 2 (SARS-CoV-2) virus (a coronavirus) in 17β-HSD12–depleted A459-ACE2 cells (fig. S3, F to H). On the other hand, the replication of hepatitis C virus (HCV), an hepatotropic flavivirus, was highly dependent on 17β-HSD12 in hepatoma cells, as reported previously (*23*) (fig. S3E). Thus, positive-strand viruses vary widely in their requirement for 17β-HSD12, even within the same virus family, suggesting broadly different requirements for lipids generated in the fatty acid elongation cycle.

To understand how 17β-HSD12 deletion affects lipid metabolism, we carried out a global lipidomics analysis of HSD12-KO54.1 cells (fig. S4C and table S2). Overall, 17β-HSD12 deletion resulted in large reductions in the relative abundance of CE, DG, and PG (fig. S4, D and E). Of 839 lipid species identified, the relative molar abundance was increased in 346 (41.3%) and decreased in 174 (20.7%) (fold change >2 or < 0.5, *Q* < 0.01) (fig. S4F). Most notably, lipids with VLCFA tails >22 carbons were sharply reduced, both overall and in each major lipid class with the exception of lysophosphatidylethanolamine (LPE) (Fig. 2, K and L, and fig. S4, G to I). Among individual lipid species, the degree of reduction correlated strongly with acyl tail length, consistent with a failure of VLCFA synthesis (Fig. 2M). Also noteworthy were large increases in lipids with OCFA tails, which represented 19.4 ± 0.3 mol % in HSD12-KO54.10 cells but only 7.1 ± 1.1 mol % in sgCtrl cells (Fig. 2N). Increases in OCFA correlated negatively with chain length (fig. S4J), reflecting the overall loss of VLCFA. PUFA were less abundant overall in HSD12-depleted cells (15.3 ± 0.47 versus 29.0 ± 0.4 mol %) (Fig. 2O), despite enrichment of many individual PUFA species (fig. S4H). Thus, the loss of HAV replication in 17β-HSD12–deleted cells can be linked to marked reductions in lipids with polyunsaturated VLCFA tails in multiple classes coupled with increases in shorter-length OCFA.

### Lipid composition of extracellular quasi-enveloped virions

**Fig. 3.**
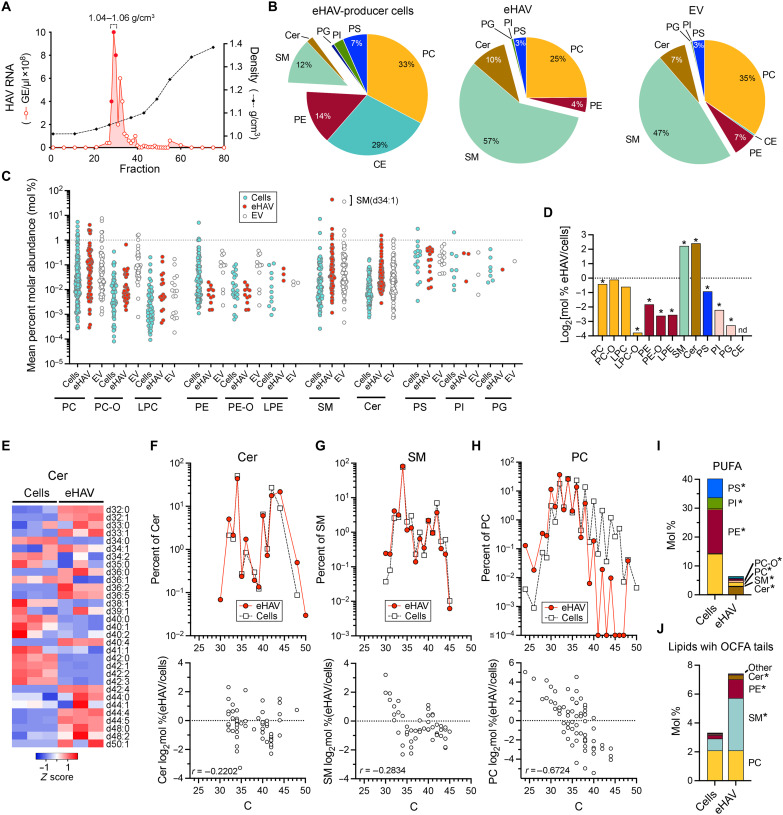
Lipid composition of extracellular eHAV and eHAV-producer cells. (**A**) Isopycnic gradient profile showing fractions containing eHAV selected for LC-MS/MS lipidomics analysis. GE = HAV genome equivalents. (**B**) Normalized percent molar abundance (mol %) of major nonglycerolipid lipid classes in eHAV-producer cells (left), eHAV (center) and control EV (right) samples. (**C**) Mean mol % abundance of individual species of major lipid classes identified in eHAV-producer cells, eHAV, or EV, ±SD, *n* = 3. SM(d34.1) abundance in eHAV and EVs is highlighted. (**D**) Enrichment of major lipid classes in eHAV versus HAV-infected producer cell samples. *Q < 0.01 by *t* test with two-stage step-up Benjamini, Krieger, and Yekutieli test. (**E**) Heatmap showing relative abundance of individual nonhexosyl Cer species identified in both eHAV and producer cells (**F**) Top: Normalized mean percent distribution of Cer species by carbon number in eHAV and producer cell samples (values shown as percent of PC). Bottom: Correlation between the fold change in relative molar abundance of individual PC species in eHAV versus producer cells, and total carbon number. *r* = Spearman correlation coefficient. *P* = 0.091. Similar analyses are shown for: (**G**) SM, *P* = 0.042; and (**H**) PC, *P* = <0.0001. (**I**) Mean normalized mol % of PUFA (≧4 double bonds) by lipid class in producer cells versus eHAV. Cholesterol esters excluded from producer cell data for comparability with eHAV. *Q < 0.01. (**J**) Means normalized mol % of lipids with odd-number carbon fatty acids (OCFAs) by lipid class in producer cells versus eHAV. *Q < 0.01; “Other” includes PS and PI which were also *Q* < 0.01.

Lipids with VLCFA tails can provide stability to membranes, particularly at points of curvature, as their acyl tails are sufficiently lengthy to bridge both leaflets in a bilayer (*20*, *24*). Thus, although VLCFA are required for genome amplification, they could also contribute to the release or stability of quasi-enveloped virus. To determine whether VLCFA are incorporated into eHAV membranes, we concentrated virus from supernatant fluids of infected cells by ultracentrifugation and purified it in isopycnic density gradients (*7*). Gradient fractions containing HAV RNA (Fig. 3A) and fractions containing nonviral extracellular vesicles (EVs) of similar density (1.04 to 1.06 gm/cm^3^) from mock-infected cells were subjected to LC-MS/MS in parallel with extracts of the related cell cultures. The summed intensities of lipids in eHAV samples were ~3-fold greater than EV samples (fig. S5A), suggesting that most lipid in eHAV samples was virus associated. A total of 368 lipid species were identified in eHAV (*n* = 366) and EV (*n* = 363) fractions (table S3) and 602 species in cell extracts (table S4). CEs were largely absent in both eHAV and EV, indicating an absence of contaminating lipoproteins. Whereas PC and CE were most abundant in cells, SM was most abundant and together with Cer accounted for more than half of the lipids identified in eHAV and EV (Fig. 3, B to D, and fig. S5B). A single SM species, SM (d34:1), comprised 43.6 mol % of lipids identified in eHAV and 36.2% in EVs (Fig. 3C). All but 28 of the lipid species identified in eHAV were also identified in cells producing these virions. These 28 species represented 3.1 to 4.3% of all lipids identified in eHAV and included highly polyunsaturated Cer species with VLCFA tails (fig. S5C).

Although the roller bottle culture conditions used to produce eHAV differed from the stationary cultures studied previously (Fig. 1), long-chain and VLCFA lipids were increased in the HAV-infected cells, as expected (fig. S5, E to G). However, with the notable exception of Cer C42 to C50 (Fig. 3, E and F, and fig. S6E), lipids with VLCFA tails were generally less abundant in eHAV than their parent producer cells, resulting in negative correlations between acyl tail length and the relative abundance in eHAV versus producer cells (Fig. 3, G and H, and fig. S6F). These results suggest no specific requirement for VLCFA other than possibly VCLFA Cer in eHAV budding and release. Other notable differences between eHAV and producer cells included a lower percentage of PUFA (6.5 mol % in eHAV versus 40.7 mol % in cells, excluding CE which were not found in eHAV) (Fig. 3I), and more SM, Cer, and PE with OCFA tails (Fig. 3J).

Nanoparticle tracking analysis revealed the mean diameter of the eHAV particles to be somewhat greater than nonviral EVs (118.3 nm versus 85.7 nm) (fig. S7A). PCA indicated a clear difference in the lipids present in these samples (fig. S7B). As in eHAV, sphingolipids comprised most of the lipids in EVs, despite lower molar abundances of many individual Cer and SM species (fig. S7C). By contrast, VLCFA PC with C ≥ 38 and ether-linked PC-O and PE-O were consistently reduced in eHAV versus EV (fig. S7C). Although a mechanistic explanation is lacking, these data point to important differences in the lipid compositions of quasi-enveloped virus and nonviral exosomes.

### Infection-induced changes in the Huh-7.5 transcriptome

**Fig. 4.**
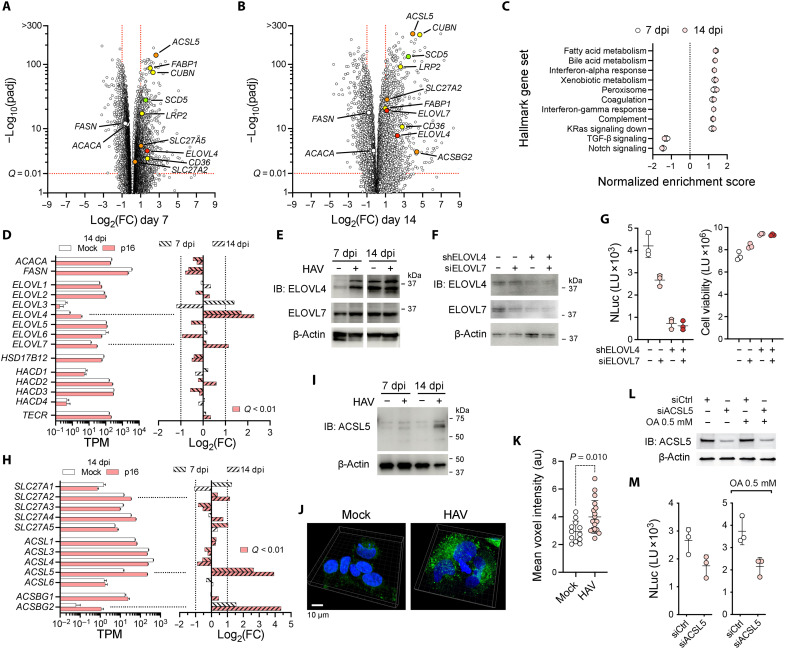
Transcriptomic analysis of p16 virus-infected cells. (**A** and **B**) Volcano plots of differentially expressed transcripts with >10 reads, fold change (FC) >2 and adjusted *P* (padj) <0.01 at (A) 7 (*n* = 1130, 823 up-regulated and 307 down-regulated) and (B) 14 dpi (*n* = 1553, 950 up-regulated and 603 down-regulated). (**C**). Normalized enrichment score (NES) of hallmark gene sets with *Q* < 0.25. (**D**) Infection-related changes in selected transcripts. Left: Bars represent transcripts per million (TPM) at14 dpi. Right: Fold change from mock-infected cells 7 and 14 dpi. Shading indicates *Q* < 0.01. (**E**) Immunoblots (IB) of ELOVL4 and ELOVL7. Mean fold increase of ELOVL4 relative to β-actin at 7 and 14 dpi was 4.0 ± 2.24 SEM, *n* = 6 blots, *P* = 0.03 (Wilcoxon sign rank test); ELOVL7, 1.5-fold ±0.37 SEM (*P* = 0.38, *n* = 4). (**F**) Immunoblots of ELOVL4 and ELOVL7 in cells transduced with nontargeting or ELOVL4-specific short hairpin RNA (shRNA), and transfected with nontargeting or ELOVL7-specific siRNA. (**G**) Left: NLuc expressed by cells shown in (F) 48 hours after infection with quasi-enveloped 18f-NLuc virus. Data from technical replicates in one of four representative experiments. Right: Cell viability assessed by ATP assay. (**H**) Long-chain acyl-CoA synthetase family transcript (left) abundance 14 dpi and (right) fold change from mock-infected cells 7 and 14 dpi. (**I**) Immunoblots of ACSL5 at 7 and 14 dpi. (**J**) Three-dimensional volume reconstruction of Airyscan fluorescence images of mock- or p16 virus–infected cells following 60 min incubation with BODIPY-C16 (1 μg/ml). (**K**) Mean cytoplasmic voxel BODIPY-C16 fluorescence intensity in mock (*n* = 271) versus infected cells (*n* = 328). *P* value by two-sided Mann-Whitney test. Data from two independent experiments. au, arbitrary units. (**L**) Immunoblots of ACSL5 in cells transfected with ACSL5-specific versus nontargeting (siCtrl) siRNA, ±0.5 mM oleic acid (OA, cis-9-octadecenoic acid, 18:1) supplementation. (**M**) NLuc expressed by 18f-NLuc virus 72 hours postinfection. Data are technical replicates from one of four independent experiments with similar results.

To identify transcriptional changes underlying alterations in lipid metabolism, we subjected RNA extracted from infected and mock-infected cells to high-throughput sequencing. An average of 2.2 × 10^7^ reads mapped to the human or HAV genome in samples collected 7 and 14 dpi (table S5), with PCA of normalized data revealing clear clustering of samples by infection status (fig. S8A). On average, 2.4 × 10^5^ reads mapped to the HAV genome 7 dpi [87 reads per million reads mapped (RPM)] and 2.7 × 10^5^ reads at 14 dpi (107 RPM). Positive-strand reads outnumbered reads mapping to the negative-strand by ~1300 to 1 (table S5). A total of 1130 transcripts were differentially expressed (fold change >2 with adjusted *P* <0.01) at 7 dpi (823 up-regulated and 307 down-regulated) (Fig. 4A) and 1553 transcripts 14 dpi (950 up-regulated and 603 down-regulated) (Fig. 4B), with good concordance between the two time points (fig. S8B). Gene Set Enrichment Analysis (GSEA) found the Hallmark Fatty Acid Metabolism, Bile Acid Metabolism, Xenobiotic Metabolism, and Peroxisome gene sets most enriched among up-regulated genes, and Hallmark Notch Signaling most enriched among down-regulated genes at both 7 and 14 dpi (Fig. 4C). Among components of the fatty acid elongation cycle (Fig. 2A), transcripts encoding the VLCFA elongase ELOVL4 were increased significantly both 7 and 14 dpi, as was ELOVL7 at 14 dpi (Fig. 4, A, B, and D). Immunoblotting revealed an average 4-fold increase in the abundance of ELOVL4, and 1.5-fold increase in ELOVL7, at 7 and 14 dpi (Fig. 4E). These fatty acid elongases catalyze the first, rate-limiting step in fatty acid elongation (Fig. 2A) and are likely responsible for increased VLCFA abundance in infected cells. Consistent with this, RNA interference (RNAi)–mediated depletion of ELOVL4, and to a lesser extent ELOVL7, reduced 18f/NLuc reporter virus replication without negatively affecting cell viability (Fig. 4, F and G).

Other changes indicated a transcriptional program broadly activating Cer synthetic pathways, including serinepalmitoyltransferases, Cer synthases, and glucosylceramidases (fig. S8C), and enhancing fatty acid import and activation. Long-chain fatty acid CoA synthetase 5 (ACSL5) transcripts were increased >6-fold at 7 dpi and >15-fold at 14 dpi (Fig. 4, A, B, and H), with increases in protein expression confirmed by immunoblotting (Fig. 4I). ACSL5 converts long-chain fatty acids to acyl-CoA thioesters, activating free fatty acids for entry into anabolic pathways and thereby promoting the uptake of exogenous long-chain fatty acids (*25*, *26*) (Fig. 2A). Consistent with this, microscopy demonstrated substantial increases in the uptake of the fluorescent dye BODIPY-C16 by infected Huh-7.5 cells (Fig. 4, J and K). RNAi-mediated depletion of ACSL5 impaired virus replication, both with and without oleic acid supplementation (Fig. 4, L and M), showing that the transcriptional induction of ACSL5 is crucial for HAV replication. Acyl-CoA synthetase bubblegum family member 2 (ACSBG2) transcripts were similarly increased but not investigated further as the overall level of expression was low (Fig. 4, B and H). Lesser increases occurred in long-chain fatty acid transport protein A2 (SLC27A2) and A5 (SLC27A5) transcripts (Fig. 4, A, B, and H). Other genes involved in fatty acid import were transcriptionally induced, including fatty acid-binding protein 1 (FABP1) and the fatty acid translocase CD36 (Fig. 4, A and B, and fig. S8D). Transcripts encoding cubilin (CUBN) and low-density lipoprotein receptor–related protein 2 (LRP2), which function together to mediate endocytosis of high-density lipoproteins (*27*), were also increased (Fig. 4, A and B, and fig. S8D). Taken collectively, these data indicate that infection activates a transcriptional program that enhances the import of long-chain fatty acids and their conversion to the activated acyl-CoA form, leading to increased VLCFA synthesis to support HAV replication.

Fatty acid synthesis is regulated within the liver by a complex regime involving the sterol response element–binding protein (SREBP) pathway and multiple ligand-activated nuclear receptors (*28*). The transcriptional control of ELOVL4 is not well defined in vertebrates, but both ACSL5 and ELOVL7 are transcriptionally regulated by SREBP-1 (*26*, *29*). However, immunoblots suggested no induction of proteolytic pathways activating SREBP-1 or SREBP-2, or increased *SREBF1* or *SREBF2* transcription in infected cells (fig. S8, E and F). Thus, the induction of ELOVL4/7 is likely related to activation of one or more nuclear receptors. Ingenuity Pathway Analysis of the RNA sequencing data suggested activation of the ligand-activated pregnane X (PXR) or retinoic acid receptor both 7 and 14 dpi (fig. S8, G and H). PXR activation has been shown to induce a SREBP-independent lipogenesis pathway in HepG2 hepatoma cells involving the multifunctional lipid transporter, CD36, stearoyl-CoA desaturase 1 (SCD1), and an unspecified long-chain free fatty acid elongase (*30*, *31*). As indicated above, CD36 was significantly up-regulated by HAV 7 and 14 dpi (Fig. 4, A and B, and fig. S8D), as was SCD5 but not the more highly expressed SCD1 (fig. S8I). PXR itself (nuclear receptor subfamily 1 group I member, encoded by *NR1l2*) was transcriptionally up-regulated 14 dpi (fig. S8I).

## DISCUSSION

Infection-related changes in host cell lipid metabolism represent an important but incompletely understood aspect of positive-strand RNA virus pathogenesis. HAV remodels the intracellular membranes of infected cells, creating a compact tubular-vesicular network thought to be the site of viral RNA synthesis (*5*, *32*, *33*). Similar membrane rearrangements can be induced by overexpressing the hepatovirus 2B or 2C protein, which associate with such membranes as well as double-stranded RNA replication intermediates in infected cells (*5*, *6*). Our data show that this remodeling of membranes is associated with profound changes in lipid metabolism leading to an increased abundance of sphingolipids and both saturated and polyunsaturated phospholipids with VLCFA tails (Fig. 1, B to H). These VLCFA are crucial for replication of the HAV genome, explaining the requirement for the 3-oxoacyl-CoA reductase, 17β-HSD12, and the fatty acid elongases, ELOVL4 and ELOVL7 (Figs. 2, D to H, and 4G). HCV, dengue virus, and other flaviviruses are also compromised by 17β-HSD12 depletion (*23*), as we confirm here (fig. S3E), but are not known to be dependent on fatty acid elongases or VLCFA with acyl tails containing >22 carbon atoms. By contrast, poliovirus, a picornavirus like HAV, replicates well with only a slight delay in 17β-HSD12–deficient cells (fig. S3C). This differing requirement for 17β-HSD12 among these two members of the same virus family may be related to morphological differences in rearrangement of intracellular membranes. The tubular-vesicular membrane network induced by HAV is more compact than that induced by poliovirus, involving membranous structures more oblong and tubular in shape (*5*). Lipids with VLCFA tails could enhance the stability of these membranes by spanning the bilayer and engaging both leaflets, thereby imparting stability that could be particularly important at points of membrane curvature (*20*, *24*). The inclusion of sphingolipids with VLCFA tails could also facilitate signal transduction across the bilayer (*34*), although it is not clear how that would contribute to replication.

While basal levels of 17β-HSD12 expression are sufficient for HAV replication, replication requires a reprogramming of transcription in Huh-7.5 cells to enhance expression of ELOVL4/7 (Fig. 4, A, B, and E). These fatty acid elongases catalyze the initial step in the fatty acid elongation cycle. Our data suggest a primary role for ELOVL4 (Fig. 4, G and F), an endoplasmic reticulum–resident glycoprotein with greater activity than ELOVL7 for VLCFA substrates with ≧26 carbons (*35*). However, ELOVL4 expression is tissue specific, highest in retina and brain, and low to negligible in liver (*36*). Thus, in vivo, HAV infection may be dependent on ELOVL7. Human cytomegalovirus (HCMV), a herpesvirus with a large DNA genome, is also dependent on VLCFA for productive infection (*37*). The HCMV envelope is enriched in C24 to C36 saturated lipids that are essential for virion infectivity. VLCFA Cer C42-50 were enriched in eHAV and could similarly enhance the infectivity of these particles (Fig. 3, E and F).

The acyl-CoA synthetase, ACSL5, was also induced by infection (Fig. 4, H and I). ACSL5 activates exogenous fatty acids for chain elongation and was essential for efficient HAV replication (Fig. 4, L and M). ACSL5 has a preference for C16 to C20 substrates, whereas other acyl-CoA synthetases induced by infection (SLC27A2 and ACSBG2) are more active for VLCFA (C20 to C24) (*38*). Like ELOVL4/7, ACSL5 transcription is regulated by both SREBP-1 and ligand-activated nuclear receptors [hepatocyte nuclear receptor 4 (HNF4)] (*39*). Overexpression of ACSL5 in rat hepatoma cells results in acylation of exogenous fatty acids and enhances fatty acid uptake, channeling fatty acids toward anabolic pathways (*40*). Consistent with this, we found that long-chain fatty acid (C16) uptake was increased in infected cells (Fig. 4, J and K). Fatty acid import is similarly increased in poliovirus-infected HeLa cells but by a distinctly different mechanism involving the activation of a different acyl-CoA synthetase family member, ACSL3, by poliovirus 2A protein (*41*).

Our data also reveal a unique dependence of the HAV life cycle on sphingolipids. Cer and SM were among the lipids most increased in abundance in infected cells and were highly enriched in the membranes of extracellular eHAV particles (Figs. 1, B and C, and 3, B to D). This enrichment likely reflects the lipid composition of the endosomal membranes from which eHAV is derived and suggests that endosomal lipid rafts enriched in sphingolipids provide a platform for the interactions of the capsid with components of endosomal sorting complexes required for transport that drive eHAV biogenesis (*8*, *10*). Cers promote endosomal intralumenal vesicle formation (*42*, *43*), and exosomes are known to be enriched in both SM and Cer (*44*, *45*), as we have confirmed here (fig. S5D). Overall similarities in the lipid composition of eHAV and EVs of similar size and buoyant density (Fig. 3, B and C) add to existing proteomics evidence that eHAV shares a mechanism of biogenesis with exosomes originating in multivesicular endosomes (*10*). The eHAV membrane contains relatively few PUFA species (Fig. 3I), a feature also described in exosomes (*42*, *45*). By contrast, we found a much lower abundance of ether-linked phospholipids (PC-O, LPC-O, and PE-O) in eHAV than in exosomes (Fig. 3C). Ether-linked phospholipids were also markedly reduced in abundance in infected cells (Fig. 1B). These lipids insert into membranes in a manner distinct from ester-linked phospholipids, influencing membrane stability and promoting the capacity for membrane fusion (*45*). This is an important point of distinction between eHAV and exosomes, as during viral entry successful delivery of the eHAV genome to the cytoplasm results from endolysosomal degradation of the eHAV membrane (*8*, *18*, *46*), whereas exosomes deliver their cargo to the cytoplasm via fusion with endosomal membranes (*47*, *48*).

## MATERIALS AND METHODS

### Viruses and plasmids

HAV stocks were generated from pHM175-p16.2 (p16 virus) (*17*) (GenBank KP879217.1) and pHM175-18 f.2 (“18f” virus) (*49*) (GenBank, KP879216.1) plasmid DNA. Naked (nHAV) and quasi-enveloped (eHAV) virus stocks were prepared from cell culture supernatants and infected cell lysates and purified by isopycnic gradient centrifugation, as described previously (*7*, *50*). Infections were carried out at a multiplicity of infection (MOI) of 1, unless specified otherwise. The nanoluciferase HAV 18f-NLuc reporter virus (*21*), poliovirus PV1-NLuc reporter virus (*51*, *52*), SARS-CoV-2–NLuc reporter virus (*53*), and *Gaussia princeps* luciferase HCV reporter viruses, H77S/GLuc2A and HJ3-5/GLuc2A virus (*54*), have been previously described.

### Cells

Human hepatoma-derived Huh-7.5 cells (*55*) and H1-HeLa were obtained from Apath LLC and the American Type Culture Collection, respectively, maintained in Dulbecco’s modified Eagle’s medium (DMEM) with 3 to 10% fetal bovine serum (FBS) and 1% penicillin/streptomycin, and tested negative for mycoplasma by polymerase chain reaction (PCR) assay (LookOut Mycoplasma PCR Detection Kit, Sigma-Aldrich). Huh-7.5 cells with depletion of ACC1 (ACC1-KO89.1 and ACC1-KO92.5 cell clones) or 17β-HSD12 (17β-HSD12–KO51.T3 and 17β-HSD12–KO54.10) were generated by CRISPR–Cas9-mediated gene editing using methods described previously (*50*). After 2 weeks culture in puromycin (6 μg ml^−1^), single-cell clones were isolated by limiting dilution, expanded in puromycin, and frozen in aliquots at −80°C. ACC1 KO89.1 and HSD12 KO54.10 cells were inoculated with 18f-NLuc nHAV for 2 hours at 37°C. Palmitic acid (200 nM), oleic acid (200 nM), or hexanoic acid (20 μM) were added to the medium. Cells were lysed and assayed for NLuc activity 72 hours postinfection. Cell viability was assessed by a luminescent assay for ATP (CellTiter-Glo, Promega #G7570).

### Reagents and antibodies

Mission short hairpin RNA (shRNA) targeting ELOVL4 (Sigma-Aldrich) and SMARTPool ON-TARGETplus small interfering RNAs (siRNAs) targeting ELOVL7 and ACSL5 (Dharmacon) were used for genetic depletion experiments. Fatty acid supplements were purchased from Sigma-Aldrich. The NLuc GLOW Assay kit (#325) was purchased from Nanolight Technology. Hoechst 33342 solution (#H3570) was obtained from Thermo Fisher Scientific. Anti-HAV capsid antibodies included mouse monoclonal antibodies K34C8 and K24F2 (Commonwealth Serum Laboratories) (*56*) and R10 (Absolute Antibody, Ab00933-1.1), and human polyclonal antibody, JC (*7*). Other antibodies included were as follows: ACC1 (Proteintech, 21923-1-AP), ACSL5 (Proteintech, 15708-1-AP, or Santa Cruz Biotechnology, sc-398310), ELOVL4 (Proteintech, 55023-1-AP), ELOVL7 (Sigma-Aldrich, SAB35500390), 17β-HSD12 (Abcam, ab236990), SREBP-1 (Novus Biologicals, NB100-2215), SREBP-2 (Abcam, ab30682), and β-actin (Sigma-Aldrich, AC74). Species-specific Alexa Fluor–conjugated secondary antibodies were from Thermo Fisher Scientific. Anti-mouse and rabbit horseradish peroxidase (HRP)–labeled secondary antibodies were from Proteintech, and IRDye 800CW donkey anti-goat immunoglobulin G antibody from Li-Cor Biosciences (#926-32214). BODIPY-C9 (#B3824) and BODIPY-C16 (#D3821) were purchased from Invitrogen. The ACC1 inhibitor firsocostat (GS-096, ND-630) was purchased from MedChemExpress (#HY-16901).

### Lentivirus production and shRNA transduction

For shRNA lentivirus production, shRNA plasmids obtained from Sigma-Aldrich were cotransfected with MISSION Lentiviral Packaging Mix (Sigma-Aldrich, #SHP001) into 293 T cells; the supernatant fluids collected at 48 and 72 hours were filtered through a 0.22-μm syringe filter. Lentiviral transduction was carried out in cells supplemented with polybrene (8 μg ml^−1^), followed by antibiotic selection with puromycin (6 μg ml^−1^).

### siRNA-mediated genetic depletion

Huh-7.5 cells were transfected with 12.5 to 50 nM SMARTPool ON-TARGETplus siRNAs (Dharmacon) using the Lipofectamine RNAiMAX transfection reagent (Thermo Fisher Scientific) according to the manufacturer’s instructions. Three to 4 days post-transfection, cells were inoculated with 18f-NLuc HAV at an MOI of ~10^3^ genome equivalents (GE) per cell. The virus was allowed to adsorb to cells for 2 hours at 37°C. The inoculum was removed, cells refed with fresh media, and incubated at 37°C until harvest.

### Cellular import of BODIPY-C16

Huh-7.5 cells infected for 8 days with p16 virus, or mock infected, were plated on glass-bottom dishes (Cellvis, D35-14-1.5-N). One day later, the cells were washed with serum-free media and incubated in serum-free media containing BODIPY-C16 (1 μg/ml) for 60 min, then washed with phosphate-buffered saline (PBS), fixed with 4% paraformaldehyde for 12 min, and washed twice more. Nuclei were counterstained with Hoechst 33342 (Invitrogen) for 10 min at room temperature. Images were collected in confocal and super-resolution modes on a laser-scanning confocal Zeiss 880 microscope (Carl Zeiss AG, Oberkochen, Germany) equipped with an Airyscan detector and controlled by Zen Blue 3.0 software. Imaris 10.0 software (Oxford Instrument) was used for quantitative analysis of the BODIPY fluorescence signal. The thre-dimensional surface of cells was rendered on the basis of the BODIPY intensity threshold for individual Z-stacks of confocal images under visual control. The mean value of BODIPY intensity for voxels below the cell surface within the cytoplasm, excluding voxels in space occupied by nuclei, was collected for each image, and analyzed statistically by two-sided Mann-Whitney test.

### HAV infections for transcriptomic and lipidomic analysis

Huh-7.5 cells were seeded at 25% confluency in T225 flasks (Corning) and inoculated 24 hours later with p16 virus at an MOI of 10 GE per cell. Following a 2-hour incubation at 37°C, the inoculum was removed and cells refed with maintenance medium (DMEM with 3% FBS). The cells were subsequently passaged every 2 to 3 days to maintain 70 to 80% confluency. Triplicate T75 flasks of infected and uninfected cells at near confluency were harvested for lipidomics (5 × 10^6^ cells) and transcriptomics analysis (1 × 10^6^ cells) 7 and 14 days after initial infection. For the lipidomics analysis, cells were washed with PBS twice, suspended in 2.5 ml of PBS in a 35-mm dish (Corning) on ice, and exposed to ultraviolet (UV) light with the lid open in a UV cross-linker for 20 min at 1200 × 100 mJ to inactivate HAV. Cells were then frozen in liquid nitrogen and stored at −80°C. For the transcriptomics analysis, RNA was extracted using the RNeasy Mini Kit (Qiagen), yielding 3 μg of total RNA. Mycoplasma infection was excluded by testing with the LookOut Mycoplasma PCR Detection Kit (Sigma-Aldrich) and HAV infection status confirmed in all samples by RT-qPCR.

### Untargeted lipidomics analysis of HAV-infected cell samples

#### Total lipid extraction

Lipids were extracted from cultured cells using the methanol/chloroform/methyl-tert-butyl-ether protocol (*57*), with minor modification. Briefly, 5 × 10^6^ cells were pelleted by low-speed centrifugation and resuspended in 600 μl of methanol:methyl-butyl ether:chloroform (4:3:3, v/v/v) mixture spiked with 10-μl mixture of 13 deuterium–labeled internal standards (EqualSPLASH Mix, Avanti Polar Lipids) at a concentration of 10 μg/ml each and homogenized using a Precellys Evolution homogenizer (Bertin Technologies) at 6800 rpm for 30 s in 2 cycles. The homogenate was vortexed for 30 s and incubated on a shaker at 900 rpm for 1 hour at 22°C. Then, the mixture was centrifuged at 17,500*g* at 22°C for 15 min and dried under a stream of nitrogen. The extracted lipids were reconstituted in 60 μl of acetonitrile:isopropanol:H_2_O (65:30:5, v/v/v), from which 10 μl was taken to prepare the pooled extract as quality control samples. All samples were stored at −20°C before analysis.

#### Reversed-phase LC-MS/MS analysis

A binary Vanquish UHPLC system (Thermo Fisher Scientific) coupled with Q Exactive HF mass spectrometer (Thermo Fisher Scientific) was used for reversed-phase LC-MS/MS (RPLC-MS/MS) analysis. For chromatographic separation, an Accucore Vanquish C18+ UHPLC column (150 μm by 2.1 mm, 1.5-μm particle size, Thermo Fisher Scientific) and the following mobile phases were used: A (50/50 acetonitrile/water) and B (88/10/2 isopropanol/acetonitrile/water), both containing 5 mM ammonium formate and 0.1% formic acid. The gradient was 10% B (1 min), 30% B (2 min), 50% B (3.5 min), 60% B (7 min), 80% B (18 min), 95% B (20 min), 100% B (22 min), 10% B (28.1 min), and column equilibration for an additional 2 min at 10% B. The column was maintained at 52°C at a flow rate of 230 μl/min. The injection volume was 4.0 μl. The QExactive HF was operated using electrospray ionization in both positive and negative ion mode for the pooled samples and in positive ionization mode for individual samples. The Orbitrap mass analyzer was operated at a resolving power of 120,000 in full-scan mode with the mass range 250 to 1200 mass/charge ratio (*m*/*z*) and of 30,000 in the top 10 data-dependent MS2 acquisition mode under higher-energy collisional dissociation (HCD) fragmentation with stepped normalized collision energy (CE 25 and 30 for positive mode; CE 20, 30, and 40 for negative mode). Precursor ion isolation window was set at 1 *m*/*z*.

#### Data processing

Raw data files of the pooled samples were processed using LipidSearch 4.2.27 (Thermo Fisher Scientific) for identification of lipid species by comparing the measured precursor *m*/*z* and MS/MS with an internal lipid species spectral library. The [M + H]^+^ or [M + NH4]^+^ adducts in positive mode and [M-H]^−^ or [M + HCOO]^−^ adducts in negative mode were considered for precursor ions. The mass tolerances of precursor ion and fragment ions were set to 5 and 10 ppm, respectively. The *m*-score threshold was 5.0. Lipids with Grade A-C were retained, which ensures that either fatty acyls or the class-specific fragment ions or both were present in the identification. Identified lipid species were further filtered using the correlation between the LC retention time (RT) and the total number of carbons in fatty acyl chains and total number of double bonds (*58*, *59*). For quantification, the intensity of identified lipids in each individual sample was extracted using Skyline (v19.1.0.193) (*60*) based on their exact *m*/*z* and RT obtained from LipidSearch. The intensities were further normalized by the ionization response factors derived from EqualSPLASH lipid standard mixture to account for differences in ionization efficiency between different lipid classes.

#### Statistical analysis

Lipid class statistical analysis was performed by unpaired *t* test of normalized percentage class means, with false discovery rate determined by the two-stage step-up Benjamini, Krieger, and Yekutieli method (*61*). PCA and hierarchical clustering were done in either Perseus (*62*) or MetaboAnalyst (www.metaboanalyst.ca/).

#### High-throughput RNA sequencing of HAV-infected cells and data analysis

RNA was extracted from Huh7.5 cells using the *mir*Vana miRNA Isolation Kit (Thermo Fisher Scientific, AM1560). Libraries were prepared using the TruSeq Stranded Total RNA Library Prep Gold Kit (Illumina) and sequenced on an Illumina HiSeq 4000 sequencer with pair-end 150-bp setting within Genewiz. Sequencing reads were aligned to the human genome hg38 with STAR 2.6 and transcripts quantified using Salmon 0.7 and Gencode v40. Differential expression analysis was carried out using default settings in the DESeq2 package in R4.0. Transcript counts were normalized using the DESeq2 internal median of ratios method. Genes with base mean read counts <10 were excluded from analysis. PCA plots were generated by the DESeq2 plotPCA routine after variance-stabilizing transformation of the read counts. Significance was adjusted for multiple comparisons using the Benjamini-Hochberg method. GSEA was carried out using normalized read counts for all genes and the GSEA software package: www.gsea-msigdb.org/gsea/index.jsp.

#### Isolation of quasi-enveloped eHAV for lipidomics analysis

Viral particles were gradient purified from supernatant fluids of roller-bottle cultures of p16 virus–infected Huh-7.5 cells using a protocol similar to that described previously (*10*). Cells were electroporated with RNA transcripts prepared from pHM175p16.2 DNA as previously described (*7*). After culturing for 2 weeks in DMEM with 10% FBS, infected and mock-infected (nontransfected) cells were subcultured in 850-cm^2^ roller bottles (Corning) in media supplemented with 25 mM Hepes and exosome-depleted FBS (Thermo Fisher Scientific). Roller bottles were placed on an Argos FlexiRoll rotating at 0.01 rpm in a 35.5°C incubator with no CO_2_. After 5 days, media were removed for storage at 4°C, the cells were refed, and roller bottles cultured for additional 5 days. Following a second and final collection, media from both infected and mock-infected cells were clarified by centrifugation twice at 10,000*g* for 30 min to remove large EVs. eHAV virions and nonviral EVs remaining in the media were concentrated by centrifugation at 100,000*g* in a Sorvall Ultra-80 ultracentrifuge with a Superspin 630 rotor and 36-ml Ultra-Clear tubes (Beckman). Pellets were resuspended in 100 μl of PBS, layered on top of a preformed preparative iodixanol gradient, and centrifuged to equilibrium as previously described (*7*). Small volume fractions were collected from the region of the gradient expected to contain virus, followed by RNA extraction and HAV-specific reverse transcription PCR to identify fractions containing virus (*7*). Fractions of equivalent density were selected for lipidomics analysis from parallel gradients loaded with material similarly concentrated from mock-infected cells. Samples of both infected and mock-infected cells were submitted in parallel for lipidomics analysis.

#### Untargeted lipidomics for eHAV samples

eHAV samples obtained from iodixanol density gradient centrifugation were transferred to total recovery glass vials (Waters) and dried with a Speedvac, followed by reconstitution in 50 μl of chloroform and sonication for 10 min to disrupt membranes. After drying, the samples were reconstituted in 30 μl of a acetonitrile:isopropanol:H_2_O (65:30:5, v/v/v) mixture for RPLC-MS/MS analysis using a method reported previously (*63*, *64*). In brief, an Accucore C30 column (150 μm by 2.1 mm, 2.6-μm particle size, Thermo Fisher Scientific) was used for lipid separation with an injection volume of 15 μl and with the gradient and MS parameters were similar to what used for the analysis of the cell lipidome. Data processing was done similarly as in the cell lipidomic studies described above, except that LipidSearch 4.1 was used for lipid identification, and peak intensities of each identified lipid species were obtained from extracted ion chromatograms in R with xcms package. Lipid class statistical analysis was by unpaired *t* test of normalized percentage class means, with false discovery rate determined by the two-stage step-up Benjamini, Krieger, and Yekutieli method (*61*).

#### Quantitative PCR with reverse transcription assay of HAV RNA

Total RNA was extracted from infected cells using the RNeasy Kit (Qiagen) and used in cDNA synthesis reactions with the SuperScript III First-Strand Synthesis System (Invitrogen). Primers targeting the 5′untranslated region of the HAV genome were used to quantify HAV RNA in a SYBR Green Real-Time qPCR assay (BioRad, #1725121) against a synthetic RNA standard as described (*50*).

#### Nanoluciferase reporter virus assay

Cells infected with18f-NLuc virus were lysed in 1× passive lysis buffer (Promega) for 5 min at room temperature. Cell lysates were transferred to an opaque white 96-well plate (Corning, 3912), mixed with 1 × Oplophorus luciferase diluted in NLuc GLOW buffer (Nanolight Technologies, 325) and incubated for 3 to 5 min at room temperature. NLuc luminescence was measured using a Biotek Synergy II multimode plate reader (BioTek Instruments).

#### SDS–polyacrylamide gel electrophoresis and immunoblotting

Approximately 10^6^ cells were lysed in radioimmunoprecipitation assay buffer (BioDynamics Laboratory, F015 or Millipore, 20-188) or Cell Lysis Buffer (Cell Signaling Technology, #9803) with a protease inhibitor (Takara, #635673) for 20 or 30 min on ice and clarified by centrifugation at 14,000*g* for 10 or 20 min at 4°C. Lysate was mixed with 4× Laemmli buffer, incubated at 95°C for 5 min, and resolved in a precast 4 to 15% (Bio-Rad, 4561086) or 10 to 20% (ATTO, 2331975) SDS–polyacrylamide gel. Proteins were transferred to a polyvinylidene fluoride membrane by semidry transfer using a Transblot Turbo apparatus (Bio-Rad). Membranes were blocked in Odyssey Blocking Buffer (LI-COR Biosciences) or PVDF Blocking Reagent for Can Get Signal (TOYOBO, NYPBR01) and probed with a 1:1000 dilution of anti-ACC1, anti-HSD17B12, anti-ACSL5, anti–SREBP-1, or anti–SREBP-2 antibodies overnight. The membrane was washed with 0.05% Tween 20 and probed with a 1:10,000 dilution of donkey anti-rabbit secondary antibodies conjugated with IR-Dye 800 (LI-COR Biosciences), 1:2000 goat anti-rabbit secondary antibodies, or 1:20,000 Western BLoT Rapid Detect v2.0 (Takara, T7122A) for 1 hour at room temperature. Excess secondary antibodies were removed by washing with 0.05% Tween 20, and protein bands were visualized using an Odyssey Infrared Imaging System (LI-COR Biosciences) or Amersham ImageQuant 800 (Cytiva) with Western BLoT Quant HRP Substrate (Takara, T7102A) or Western BLoT Ultra Sensitive HRP Substrate (Takara, T7104A).

## Supplementary Materials

### This PDF file includes:

Figs. S1 to S8 Legends for tables S1 to S5 References

## References

[1] S. M. Lemon, J. J. Ott, P. Van Damme, D. Shouval, Type A viral hepatitis: A summary and update on the molecular virology, epidemiology, pathogenesis and prevention. J. Hepatol. 68, 167–184 (2018). 10.1016/j.jhep.2017.08.034 28887164

[2] K. H. Jacobsen, Globalization and the changing epidemiology of hepatitis A virus. Cold Spring Harbor. Perspect. Med. 8, a031716 (2018). 10.1101/cshperspect.a031716 PMC6169986 29500305

[3] K. L. McKnight, S. M. Lemon, Hepatitis A virus genome organization and replication strategy. Cold Spring Harbor. Perspect. Med. 8, a033480 (2018). 10.1101/cshperspect.a033480 PMC6280712 29610147

[4] D. B. Smith, P. Simmonds, Classification and genomic diversity of enterically transmitted hepatitis viruses. Cold Spring Harbor. Perspect. Med. 8, a031880 (2018). 10.1101/cshperspect.a031880 PMC6120691 29530950

[5] R. Gosert, D. Egger, K. Bienz, A cytopathic and a cell culture adapted hepatitis A virus strain differ in cell killing but not in intracellular membrane rearrangements. Virology 266, 157–169 (2000). 10612670 10.1006/viro.1999.0070

[6] N. L. Teterina, K. Bienz, D. Egger, A. E. Gorbalenya, E. Ehrenfeld, Induction of intracellular membrane rearrangements by HAV proteins 2C and 2BC. Virology 237, 66–77 (1997). 9344908 10.1006/viro.1997.8775

[7] Z. Feng, L. Hensley, K. L. McKnight, F. Hu, V. Madden, L. Ping, S.-H. Jeong, C. Walker, R. E. Lanford, S. M. Lemon, A pathogenic picornavirus acquires an envelope by hijacking cellular membranes. Nature 496, 367–371 (2013). 23542590 10.1038/nature12029 PMC3631468

[8] A. Das, E. E. Rivera-Serrano, X. Yin, C. M. Walker, Z. Feng, S. M. Lemon, Cell entry and release of quasi-enveloped human hepatitis viruses. Nat. Rev. Microbiol. 21, 573–589 (2023). 37185947 10.1038/s41579-023-00889-z PMC10127183

[9] A. Hirai-Yuki, L. Hensley, J. K. Whitmire, S. M. Lemon, Biliary secretion of quasi-enveloped human hepatitis A virus. mBio 7, e01998–e01916 (2016). 27923925 10.1128/mBio.01998-16 PMC5142623

[10] K. L. McKnight, L. Xie, O. González-López, X. Chen, S. M. Lemon, Protein composition of the hepatitis A virus quasi-envelope. Proc. Natl. Acad. Sci. U.S.A. 114, 6587–6592 (2017). 28490497 10.1073/pnas.1619519114 PMC5488923

[11] Y.-H. Chen, W. L. Du, M. C. Hagemeijer, P. M. Takvorian, C. Pau, A. Cali, C. A. Brantner, E. S. Stempinski, P. S. Connelly, H.-C. Ma, P. Jiang, E. Wimmer, G. Altan-Bonnet, N. Altan-Bonnet, Phosphatidylserine vesicles enable efficient en bloc transmission of enteroviruses. Cell 160, 619–630 (2015). 25679758 10.1016/j.cell.2015.01.032 PMC6704014

[12] M. Santiana, S. Ghosh, B. A. Ho, V. Rajasekaran, W.-L. Du, Y. Mutsafi, D. A. De Jésus-Diaz, S. V. Sosnovtsev, E. A. Levenson, G. I. Parra, P. M. Takvorian, A. Cali, C. Bleck, A. N. Vlasova, L. J. Saif, J. T. Patton, P. Lopalco, A. Corcelli, K. Y. Green, N. Altan-Bonnet, Vesicle-cloaked virus clusters are optimal units for inter-organismal viral transmission. Cell Host Microbe 24, 208–220.e8 (2018). 30092198 10.1016/j.chom.2018.07.006 PMC6226266

[13] J. Morris-Love, G. V. Gee, B. A. O’Hara, B. Assetta, A. L. Atkinson, A. S. Dugan, S. A. Haley, W. J. Atwood, M. Imperiale, D. Galloway, JC polyomavirus uses extracellular vesicles to infect target cells. MBio 10, e00379-19 (2019). 30967463 10.1128/mBio.00379-19 PMC6456752

[14] S. G. van der Grein, K. A. Y. Defourny, H. H. Rabouw, S. S. Goerdayal, M. J. C. van Herwijnen, R. W. Wubbolts, M. Altelaar, F. J. M. van Kuppeveld, E. N. M. Nolte-‘t Hoen, The encephalomyocarditis virus Leader promotes the release of virions inside extracellular vesicles via the induction of secretory autophagy. Nat. Commun. 13, 3625 (2022). 35750662 10.1038/s41467-022-31181-y PMC9232559

[15] X. Han, R. W. Gross, The foundations and development of lipidomics. J. Lipid Res. 63, 100164 (2022). 34953866 10.1016/j.jlr.2021.100164 PMC8953652

[16] T. Züllig, H. C. Köfeler, High resolution mass spectrometry in lipidomics. Mass Spectrom. Rev. 40, 162–176 (2021). 32233039 10.1002/mas.21627 PMC8049033

[17] R. W. Jansen, J. E. Newbold, S. M. Lemon, Complete nucleotide sequence of a cell culture-adapted variant of hepatitis A virus: Comparison with wild-type virus with restricted capacity for in vitro replication. Virology 163, 299–307 (1988). 2833008 10.1016/0042-6822(88)90270-x

[18] A. Das, R. Barrientos, T. Shiota, V. Madigan, I. Misumi, K. L. McKnight, L. Sun, Z. Li, R. M. Meganck, Y. Li, E. Kaluzna, A. Asokan, J. K. Whitmire, M. Kapustina, Q. Zhang, S. M. Lemon, Gangliosides are essential endosomal receptors for quasi-enveloped and naked hepatitis A virus. Nat. Microbiol. 5, 1069–1078 (2020). 32451473 10.1038/s41564-020-0727-8 PMC7483933

[19] J. K. Hiltunen, A. J. Kastaniotis, K. J. Autio, G. Jiang, Z. Chen, T. Glumoff, 17B-hydroxysteroid dehydrogenases as acyl thioester metabolizing enzymes. Mol. Cell. Endocrinol. 489, 107–118 (2019). 30508570 10.1016/j.mce.2018.11.012

[20] A. Kihara, Very long-chain fatty acids: elongation, physiology and related disorders. J. Biochem. 152, 387–395 (2012). 22984005 10.1093/jb/mvs105

[21] D. Yamane, H. Feng, E. E. Rivera-Serrano, S. R. Selitsky, A. Hirai-Yuki, A. das, K. L. McKnight, I. Misumi, L. Hensley, W. Lovell, O. González-López, R. Suzuki, M. Matsuda, H. Nakanishi, T. Ohto-Nakanishi, T. Hishiki, E. Wauthier, T. Oikawa, K. Morita, L. M. Reid, P. Sethupathy, M. Kohara, J. K. Whitmire, S. M. Lemon, Basal expression of interferon regulatory factor 1 drives intrinsic hepatocyte resistance to multiple RNA viruses. Nat. Microbiol. 4, 1096–1104 (2019). 30988429 10.1038/s41564-019-0425-6 PMC6588457

[22] M. Yi, S. M. Lemon, Replication of subgenomic hepatitis A virus RNAs expressing firefly luciferase is enhanced by mutations associated with adaptation of virus to growth in cultured cells. J. Virol. 76, 1171–1180 (2002). 11773393 10.1128/JVI.76.3.1171-1180.2002 PMC135777

[23] B. Mohamed, C. Mazeaud, M. Baril, D. Poirier, A. A. Sow, L. Chatel-Chaix, V. Titorenko, D. Lamarre, Very-long-chain fatty acid metabolic capacity of 17-beta-hydroxysteroid dehydrogenase type 12 (HSD17B12) promotes replication of hepatitis C virus and related flaviviruses. Sci. Rep. 10, 4040 (2020). 32132633 10.1038/s41598-020-61051-w PMC7055353

[24] T. Róg, A. Orłowski, A. Llorente, T. Skotland, T. Sylvänne, D. Kauhanen, K. Ekroos, K. Sandvig, I. Vattulainen, Interdigitation of long-chain sphingomyelin induces coupling of membrane leaflets in a cholesterol dependent manner. Biochim. Biophys. Acta 1858, 281–288 (2016). 26654782 10.1016/j.bbamem.2015.12.003

[25] S. Y. Bu, D. G. Mashek, Hepatic long-chain acyl-CoA synthetase 5 mediates fatty acid channeling between anabolic and catabolic pathways. J. Lipid Res. 51, 3270–3280 (2010). 20798351 10.1194/jlr.M009407 PMC2952567

[26] Q. Luo, A. das, F. Oldoni, P. Wu, J. Wang, F. Luo, Z. Fang, Role of ACSL5 in fatty acid metabolism. Heliyon 9, e13316 (2023). 36816310 10.1016/j.heliyon.2023.e13316 PMC9932481

[27] R. Kozyraki, J. Fyfe, M. Kristiansen, C. Gerdes, C. Jacobsen, S. Cui, E. I. Christensen, M. Aminoff, A. de la Chapelle, R. Krahe, P. J. Verroust, S. K. Moestrup, The intrinsic factor-vitamin B12 receptor, cubilin, is a high-affinity apolipoprotein A-I receptor facilitating endocytosis of high-density lipoprotein. Nat. Med. 5, 656–661 (1999). 10371504 10.1038/9504

[28] J. Ye, R. A. DeBose-Boyd, Regulation of cholesterol and fatty acid synthesis. Cold Spring Harb. Perspect. Biol. 3, a004754 (2011). 21504873 10.1101/cshperspect.a004754 PMC3119913

[29] J. G. Purdy, T. Shenk, J. D. Rabinowitz, Fatty acid elongase 7 catalyzes lipidome remodeling essential for human cytomegalovirus replication. Cell Rep. 10, 1375–1385 (2015). 25732827 10.1016/j.celrep.2015.02.003 PMC4354725

[30] J. Zhou, Y. Zhai, Y. Mu, H. Gong, H. Uppal, D. Toma, S. Ren, R. M. Evans, W. Xie, A novel pregnane X receptor-mediated and sterol regulatory element-binding protein-independent lipogenic pathway. J. Biol. Chem. 281, 15013–15020 (2006). 16556603 10.1074/jbc.M511116200 PMC4109972

[31] J. Zhang, Y. Wei, B. Hu, M. Huang, W. Xie, Y. Zhai, Activation of human stearoyl-coenzyme A desaturase 1 contributes to the lipogenic effect of PXR in HepG2 cells. PLOS ONE 8, e67959 (2013). 23874477 10.1371/journal.pone.0067959 PMC3706516

[32] M. W. Cho, N. Teterina, D. Egger, K. Bienz, E. Ehrenfeld, Membrane rearrangement and vesicle induction by recombinant poliovirus 2C and 2BC in human cells. Virology 202, 129–145 (1994). 8009827 10.1006/viro.1994.1329

[33] D. Paul, S. Hoppe, G. Saher, J. Krijnse-Locker, R. Bartenschlager, Morphological and biochemical characterization of the membranous hepatitis C virus replication compartment. J. Virol. 87, 10612–10627 (2013). 23885072 10.1128/JVI.01370-13 PMC3807400

[34] K. Iwabuchi, H. Nakayama, C. Iwahara, K. Takamori, Significance of glycosphingolipid fatty acid chain length on membrane microdomain-mediated signal transduction. FEBS Lett. 584, 1642–1652 (2010). 19852959 10.1016/j.febslet.2009.10.043

[35] Y. Ohno, S. Suto, M. Yamanaka, Y. Mizutani, S. Mitsutake, Y. Igarashi, T. Sassa, A. Kihara, ELOVL1 production of C24 acyl-CoAs is linked to C24 sphingolipid synthesis. Proc. Natl. Acad. Sci. U.S.A. 107, 18439–18444 (2010). 20937905 10.1073/pnas.1005572107 PMC2973002

[36] M. P. Agbaga, S. Logan, R. S. Brush, R. E. Anderson, Biosynthesis of very long-chain polyunsaturated fatty acids in hepatocytes expressing ELOVL4. Adv. Exp. Med. Biol. 801, 631–636 (2014). 24664752 10.1007/978-1-4614-3209-8_79

[37] E. Koyuncu, J. G. Purdy, J. D. Rabinowitz, T. Shenk, Saturated very long chain fatty acids are required for the production of infectious human cytomegalovirus progeny. PLOS Pathog. 9, e1003333 (2013). 23696731 10.1371/journal.ppat.1003333 PMC3656100

[38] P. Fraisl, H. Tanaka, S. Forss-Petter, H. Lassmann, Y. Nishimune, J. Berger, A novel mammalian bubblegum-related acyl-CoA synthetase restricted to testes and possibly involved in spermatogenesis. Arch. Biochem. Biophys. 451, 23–33 (2006). 16762313 10.1016/j.abb.2006.04.013

[39] L. Chen, R. P. Vasoya, N. H. Toke, A. Parthasarathy, S. Luo, E. Chiles, J. Flores, N. Gao, E. M. Bonder, X. Su, M. P. Verzi, HNF4 regulates fatty acid oxidation and is required for renewal of intestinal stem cells in mice. Gastroenterology 158, 985–999.e9 (2020). 31759926 10.1053/j.gastro.2019.11.031 PMC7062567

[40] D. G. Mashek, M. A. McKenzie, C. G. Van Horn, R. A. Coleman, Rat long chain acyl-CoA synthetase 5 increases fatty acid uptake and partitioning to cellular triacylglycerol in McArdle-RH7777 cells. J. Biol. Chem. 281, 945–950 (2006). 16263710 10.1074/jbc.M507646200

[41] J. A. Nchoutmboube, E. G. Viktorova, A. J. Scott, L. A. Ford, Z. Pei, P. A. Watkins, R. K. Ernst, G. A. Belov, Increased long chain acyl-Coa synthetase activity and fatty acid import is linked to membrane synthesis for development of picornavirus replication organelles. PLOS Pathog. 9, e1003401 (2013). 23762027 10.1371/journal.ppat.1003401 PMC3675155

[42] K. Trajkovic, C. Hsu, S. Chiantia, L. Rajendran, D. Wenzel, F. Wieland, P. Schwille, B. Brügger, M. Simons, Ceramide triggers budding of exosome vesicles into multivesicular endosomes. Science 319, 1244–1247 (2008). 18309083 10.1126/science.1153124

[43] K. Yuyama, H. Sun, D. Mikami, T. Mioka, K. Mukai, Y. Igarashi, Lysosomal-associated transmembrane protein 4B regulates ceramide-induced exosome release. FASEB J. 34, 16022–16033 (2020). 33090522 10.1096/fj.202001599R

[44] T. Skotland, K. Sagini, K. Sandvig, A. Llorente, An emerging focus on lipids in extracellular vesicles. Adv. Drug Deliv. Rev. 159, 308–321 (2020). 32151658 10.1016/j.addr.2020.03.002

[45] T. Skotland, N. P. Hessvik, K. Sandvig, A. Llorente, Exosomal lipid composition and the role of ether lipids and phosphoinositides in exosome biology. J. Lipid Res. 60, 9–18 (2019). 30076207 10.1194/jlr.R084343 PMC6314266

[46] E. E. Rivera-Serrano, O. Gonzalez-Lopez, A. Das, S. M. Lemon, Cellular entry and uncoating of naked and quasi-enveloped human hepatoviruses. eLife 8, e43983 (2019). 30801249 10.7554/eLife.43983 PMC6422491

[47] E. Bonsergent, E. Grisard, J. Buchrieser, O. Schwartz, C. Théry, G. Lavieu, Quantitative characterization of extracellular vesicle uptake and content delivery within mammalian cells. Nat. Commun. 12, 1864 (2021). 33767144 10.1038/s41467-021-22126-y PMC7994380

[48] M. I. Morandi, P. Busko, E. Ozer-Partuk, S. Khan, G. Zarfati, Y. Elbaz-Alon, P. Abou Karam, T. Napso Shogan, L. Ginini, Z. Gil, N. Regev-Rudzki, O. Avinoam, Extracellular vesicle fusion visualized by cryo-electron microscopy. PNAS Nexus 1, pgac156 (2022). 36714848 10.1093/pnasnexus/pgac156 PMC9802263

[49] S. M. Lemon, P. C. Murphy, P. A. Shields, L. H. Ping, S. M. Feinstone, T. Cromeans, R. W. Jansen, Antigenic and genetic variation in cytopathic hepatitis A virus variants arising during persistent infection: evidence for genetic recombination. J. Virol. 65, 2056–2065 (1991). 1705995 10.1128/jvi.65.4.2056-2065.1991 PMC240056

[50] A. Das, A. Hirai-Yuki, O. González-López, B. Rhein, S. Moller-Tank, R. Brouillette, L. Hensley, I. Misumi, W. Lovell, J. M. Cullen, J. K. Whitmire, W. Maury, S. M. Lemon, TIM1 (HAVCR1) Is not essential for cellular entry of either quasi-enveloped or naked hepatitis A virions. MBio 8, e00969-17 (2017). 28874468 10.1128/mBio.00969-17 PMC5587907

[51] Y. Li, I. Misumi, T. Shiota, L. Sun, E. M. Lenarcic, H. Kim, T. Shirasaki, A. Hertel-Wulff, T. Tibbs, J. E. Mitchell, K. L. McKnight, C. E. Cameron, N. J. Moorman, D. R. McGivern, J. M. Cullen, J. K. Whitmire, S. M. Lemon, The ZCCHC14/TENT4 complex is required for hepatitis A virus RNA synthesis. Proc. Natl. Acad. Sci. U.S.A. 119, e2204511119 (2022). 35867748 10.1073/pnas.2204511119 PMC9282228

[52] H. Kim, D. Aponte-Diaz, M. S. Sotoudegan, D. Shengjuler, J. J. Arnold, C. E. Cameron, The enterovirus genome can be translated in an IRES-independent manner that requires the initiation factors eIF2A/eIF2D. PLOS Biol. 21, e3001693 (2023). 36689548 10.1371/journal.pbio.3001693 PMC9894558

[53] A. J. Pruijssers, A. S. George, A. Schäfer, S. R. Leist, L. E. Gralinksi, K. H. Dinnon III, B. L. Yount, M. L. Agostini, L. J. Stevens, J. D. Chappell, X. Lu, T. M. Hughes, K. Gully, D. R. Martinez, A. J. Brown, R. L. Graham, J. K. Perry, V. du Pont, J. Pitts, B. Ma, D. Babusis, E. Murakami, J. Y. Feng, J. P. Bilello, D. P. Porter, T. Cihlar, R. S. Baric, M. R. Denison, T. P. Sheahan, Remdesivir inhibits SARS-CoV-2 in human lung cells and chimeric SARS-CoV expressing the SARS-CoV-2 RNA polymerase in mice. Cell Rep. 32, 107940 (2020). 32668216 10.1016/j.celrep.2020.107940 PMC7340027

[54] D. Yamane, D. R. McGivern, E. Wauthier, M. K. Yi, V. J. Madden, C. Welsch, I. Antes, Y. Wen, P. E. Chugh, C. E. McGee, D. G. Widman, I. Misumi, S. Bandyopadhyay, S. Kim, T. Shimakami, T. Oikawa, J. K. Whitmire, M. T. Heise, D. P. Dittmer, C. C. Kao, S. M. Pitson, A. H. Merrill Jr., L. M. Reid, S. M. Lemon, Regulation of the hepatitis C virus RNA replicase by endogenous lipid peroxidation. Nat. Med. 20, 927–935 (2014). 25064127 10.1038/nm.3610 PMC4126843

[55] K. J. Blight, J. A. McKeating, C. M. Rice, Highly permissive cell lines for subgenomic and genomic hepatitis C virus RNA replication. J. Virol. 76, 13001–13014 (2002). 12438626 10.1128/JVI.76.24.13001-13014.2002 PMC136668

[56] A. MacGregor, M. Kornitschuk, J. G. Hurrell, N. I. Lehmann, A. G. Coulepis, S. A. Locarnini, I. D. Gust, Monoclonal antibodies against hepatitis A virus. J. Clin. Microbiol. 18, 1237–1243 (1983). 6315771 10.1128/jcm.18.5.1237-1243.1983 PMC272872

[57] R. M. Pellegrino, A. Di Veroli, A. Valeri, L. Goracci, G. Cruciani, LC/MS lipid profiling from human serum: A new method for global lipid extraction. Anal. Bioanal. Chem. 406, 7937–7948 (2014). 25381612 10.1007/s00216-014-8255-0

[58] N. Vu, M. Narvaez-Rivas, G. Y. Chen, M. J. Rewers, Q. Zhang, Accurate mass and retention time library of serum lipids for type 1 diabetes research. Anal. Bioanal. Chem. 411, 5937–5949 (2019). 31280478 10.1007/s00216-019-01997-7 PMC6762021

[59] Z. Li, Q. Zhang, Ganglioside isomer analysis using ion polarity switching liquid chromatography-tandem mass spectrometry. Anal. Bioanal. Chem. 413, 3269–3279 (2021). 33686479 10.1007/s00216-021-03262-2 PMC8672327

[60] B. MacLean, D. M. Tomazela, N. Shulman, M. Chambers, G. L. Finney, B. Frewen, R. Kern, D. L. Tabb, D. C. Liebler, M. J. MacCoss, Skyline: an open source document editor for creating and analyzing targeted proteomics experiments. Bioinformatics 26, 966–968 (2010). 20147306 10.1093/bioinformatics/btq054 PMC2844992

[61] Y. Benjamini, A. Krieger, D. Yekutieli, Adaptive linear step-up procedures that control the false discovery rate. Biometrika 93, 491–507 (2006).

[62] S. Tyanova, J. Cox, Perseus: A bioinformatics platform for integrative analysis of proteomics data in cancer research. Methods Mol. Biol. 1711, 133–148 (2018). 29344888 10.1007/978-1-4939-7493-1_7

[63] M. Narvaez-Rivas, N. Vu, G. Y. Chen, Q. Zhang, Off-line mixed-mode liquid chromatography coupled with reversed phase high performance liquid chromatography-high resolution mass spectrometry to improve coverage in lipidomics analysis. Anal. Chim. Acta 954, 140–150 (2017). 28081809 10.1016/j.aca.2016.12.003 PMC5260842

[64] M. Narváez-Rivas, Q. Zhang, Comprehensive untargeted lipidomic analysis using core-shell C30 particle column and high field orbitrap mass spectrometer. J. Chromatogr. A 1440, 123–134 (2016). 26928874 10.1016/j.chroma.2016.02.054 PMC4792668

[65] S. Babicki, D. Arndt, A. Marcu, Y. Liang, J. R. Grant, A. Maciejewski, D. S. Wishart, Heatmapper: Web-enabled heat mapping for all. Nucleic Acids Res. 44, W147–W153 (2016). 27190236 10.1093/nar/gkw419 PMC4987948

