# Supplementary material for: Hepatoviruses promote very-long-chain fatty acid and sphingolipid synthesis for viral RNA replication and quasi-enveloped virus release
Source: Sci Adv. 2023 Oct 20;9(42):eadj4198. doi: 10.1126/sciadv.adj4198 (PMC10588952; doi:10.1126/sciadv.adj4198)
Supplement: Supplementary file 1 — Figs. S1 to S8 Legends for tables S1 to S5 References [file sciadv.adj4198_sm.pdf]

Science Advances
AAAS

Supplementary Materials for

Hepatoviruses promote very-long-chain fatty acid and sphingolipid synthesis for viral RNA replication and quasi-enveloped virus release

Tomoyuki Shiota *et al.*

Corresponding author: Stanley M. Lemon, smlemon@med.unc.edu

Sci. Adv. **9**, eadj4198 (2023)
DOI: 10.1126/sciadv.adj4198

The PDF file includes:

Figs. S1 to S8
Legends for tables S1 to S5
References

Other Supplementary Material for this manuscript includes the following:

Tables S1 to S5

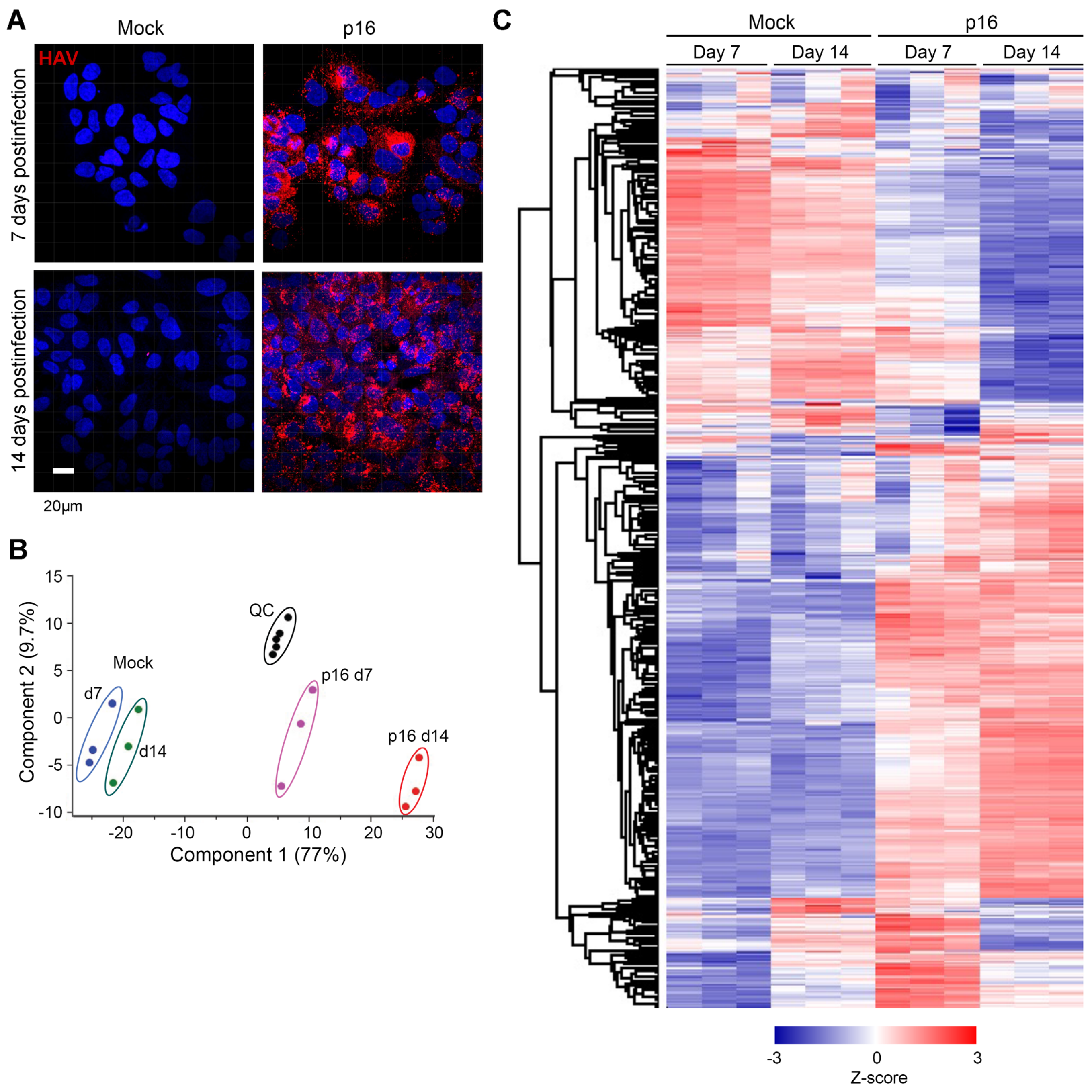

Figure S1. Global lipidomics analysis of p16 virus-infected Huh-7.5 cells. (A) Maximum projection confocal immunofluorescence microscopy images of p16 virus- and mock-infected cells 7 and 14 days postinfection (dpi). Cells were labelled (red) with JC polyclonal human anti-HAV antibody; nuclei were counterstained (blue) with Hoescht 33342. Bar, 20 µm. **(B)** Principal component analysis of normalized mass spectrometry data from triplicate cell samples collected 7 (d7) and 14 (d14) days after p16 or mock virus infection. QC, quality control samples. (**C**) Heat map showing hierarchical clustering of normalized data from all 688 lipid species identified. Overall, infection altered the relative molar abundance of 62 lipid species by 7 dpi, and 292 lipid species by 14 dpi (Q<0.01).

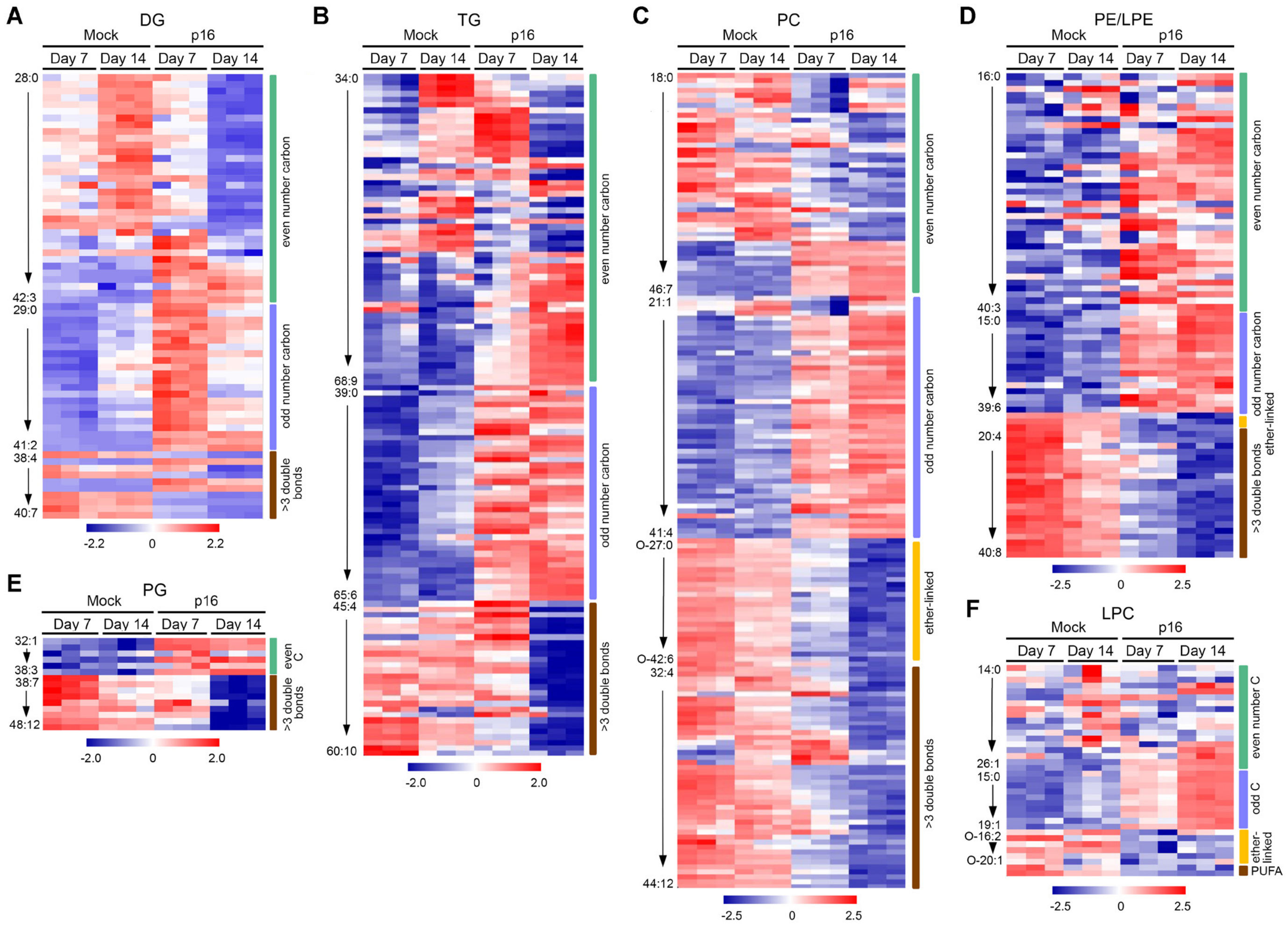

Figure S2. Heat maps of relative molar abundance of individual lipid species. Data shown for major lipid classes present in triplicate samples from mock versus p16 virus-infected Huh-7.5 cells at 7 and 14 days post-infection. **(A)** Diglycerides (DG); **(B)** Triglycerides (TG); **(C)** Phosphatidylcholine (PC); **(D)** Phosphatidylethanolamine (PE) and Lysophosphatidyl-ethanolamine (LPE); **(E)** Phosphatidylglycerol (PG); and **(F)** Lysophosphatidylcholine. Within each class, lipid species are grouped by ascending total numbers of carbon atoms, even versus odd total numbers of carbons, and the presence of >3 double bonds (PUFA). Z-score range is shown below each heat map.

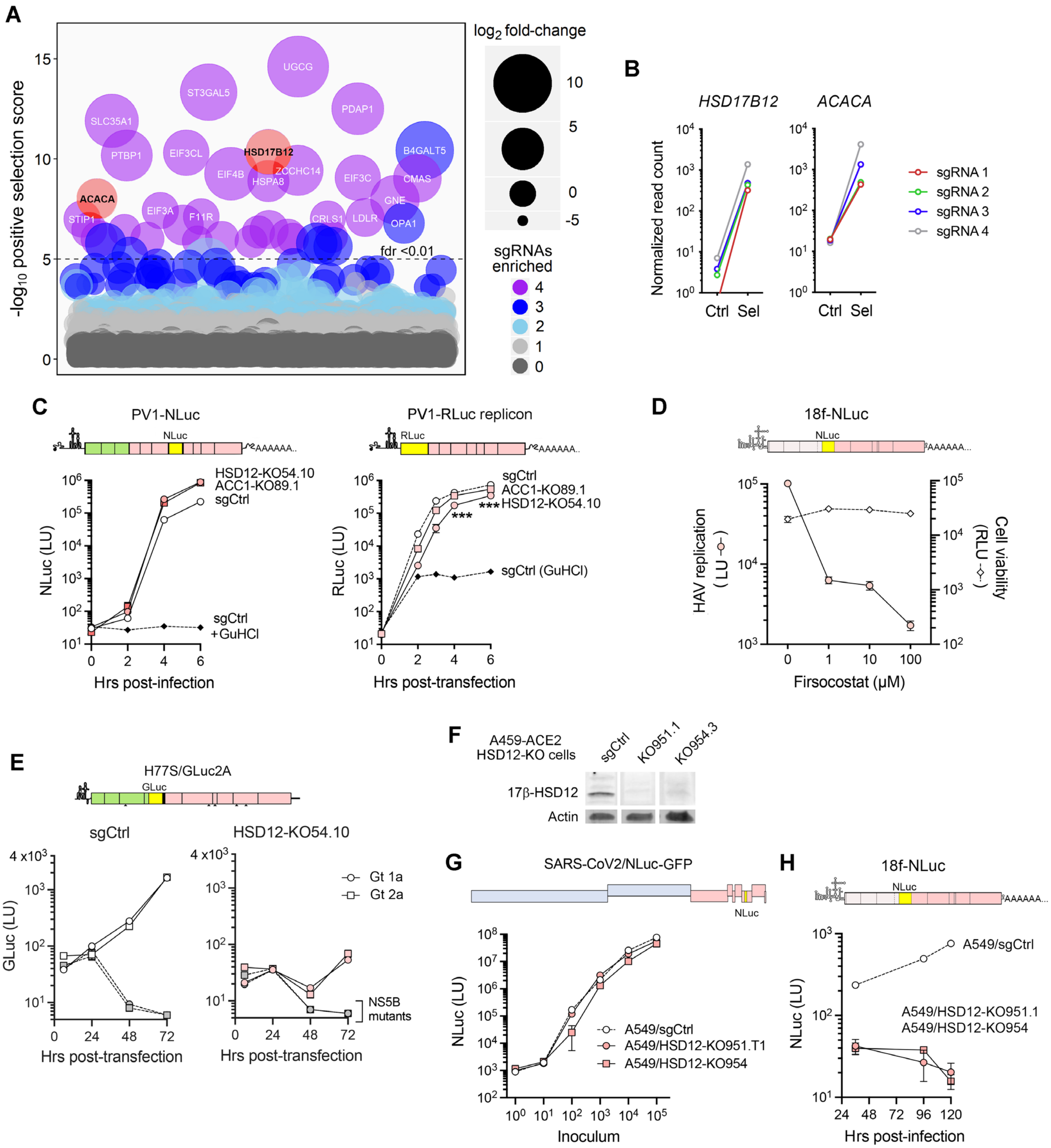

Figure S3. Positive-strand RNA virus host factor requirements for ACC1 and 17β-HSD12. (**A**) Bubble plot showing genes identified as candidate HAV host factors in a genome-wide CRISPR screen, highlighting the selection of sgRNAs targeting ACACA (encoding ACC1) and HSD17B12 (17β-HSD12). Data shown are derived from an analysis of two independent experiments and have been published previously (*18*). (**B**) Selection of sgRNAs targeting *ACACA* and *HSD17B12* (4 sgRNAs each) in the CRISPR screen. (**C**) Reporter protein expression from

(*left*) PV1-NLuc reporter virus and (*right*) PV1-RLuc replicon RNA in ACC1- and 17β-HSD12-depleted cells versus control sgCtrl with or without treatment with 3mM guanidine HCl (GuHCl). (LU, light units). (**D**) Inhibition of HAV 18f-NLuc reporter virus replication by the ACC1 inhibitor Firsocostat. Cells were pretreated with the compound overnight, then infected and lysed 72 hrs later for NLuc assay. Cell viability was assessed by assay for ATP activity with results (RLU, relative light units) presented on the right axis. **(E)** Reporter protein expressed by genotype (gt) 1a H77S/GLuc2A and gt 2a HJ3-5/GLuc2A hepatitis C virus (HCV) RNA following transfection of HSD12-KO54.10 or sgCtrl cells. Cells were transfected in parallel with replication-incompetent viral RNAs with mutations in the NS5B RNA-dependent RNA polymerase (H77S/GLuc2A-AAG or HJ3-5/GLuc2A-GND). (**F)** Immunoblots of 17β-HSD12 in lysates of A549-ACE2 cells with targeted deletion of *HSD17B12*. sgCtrl cells were transduced in parallel with a nontargeting sgRNA. A549-ACE2 cells constitutively express the ACE2 coronavirus receptor. **(G)** (*top*) SARS-CoV2-NLuc/GFP reporter virus genome. (*bottom*) NLuc expressed by A549 cells with targeted deletion of 17β-HSD12 (A549/HSD12-KO951.1 or A549/HSD12-KO954) or control A549/sgCtrl cells. (**H)** NLuc expressed by 17β-HSD12-depleted A549-ACE2 cells following infection with 18f-NLuc virus.

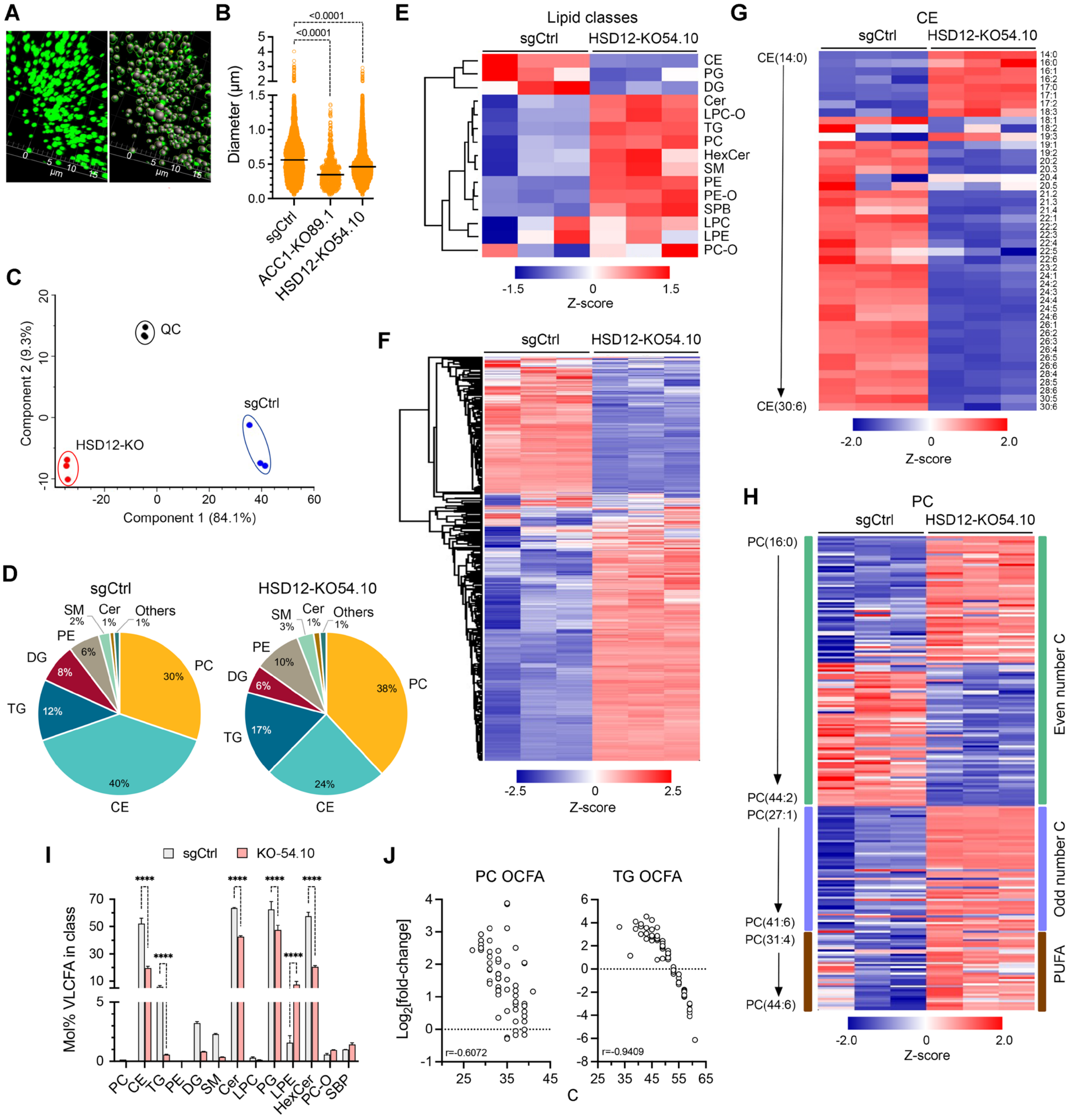

Figure S4. Lipid species in ACC1- and 17β-HSD12-depleted cells. **(A)** (*left*) Airyscan Z-stack image and (*right*) related 3D volume reconstruction of a region in the cytoplasm of BODIPY-C9-stained HSD12-KO54.10 cells. Spherical representation of lipid droplets was based on size, position, and intensity of BODIPY-C9 signals. **(B)** Estimated diameters and numbers of individual lipid droplets in: sgCtrl cells, diameter 0.562 µm (n=7,729, 236/cell); ACC1-KO89.1 cells: diameter 0.349 µm (n=988, 22/cell); HSD12-KO54.10 cells, diameter 0.462 µm (n=11,727, 274/cell). p-values by Mann-Whitney test. **(C)** Principal component analysis of normalized mass spectrometry data from triplicate samples of HSD12-KO54.10 and control sgCtrl cells. **(D)** Molar abundance of major lipid classes in sgCtrl versus HSD12-KO54.10 cells. **(E)** Heat map showing relative molar abundance of 15 major lipid classes. Clustering by Spearman rank order correlation (*65*). **(F)** Heat map showing hierarchical clustering of normalized data from all 839 lipid species

identified. (**G)** Heat map showing relative molar abundance of different cholesterol esters (CE) in the two cell types, with clustering based on acyl chain carbon number. (**H)** Heat map showing relative molar abundances of individual phosphatidylcholine (PC) species in HSD12-KO54.10 versus sgCtrl cells. Lipid species are grouped by ascending acyl chain carbon numbers, even versus odd total numbers of carbons, and the presence of >3 double bonds (PUFA). (**I)** Relative molar abundance of lipids with VLCFA tails in major lipid classes in HSD12-KO54.10 and sgCtrl cells. **(J)** Fold-change in relative molar abundance of PC and TG species with odd number of carbons between HSD12-KO54.10 and sgCtrl cells plotted against total acyl chain carbon number. r=Spearman correlation coefficient; p<0.001 for both PC and TG.

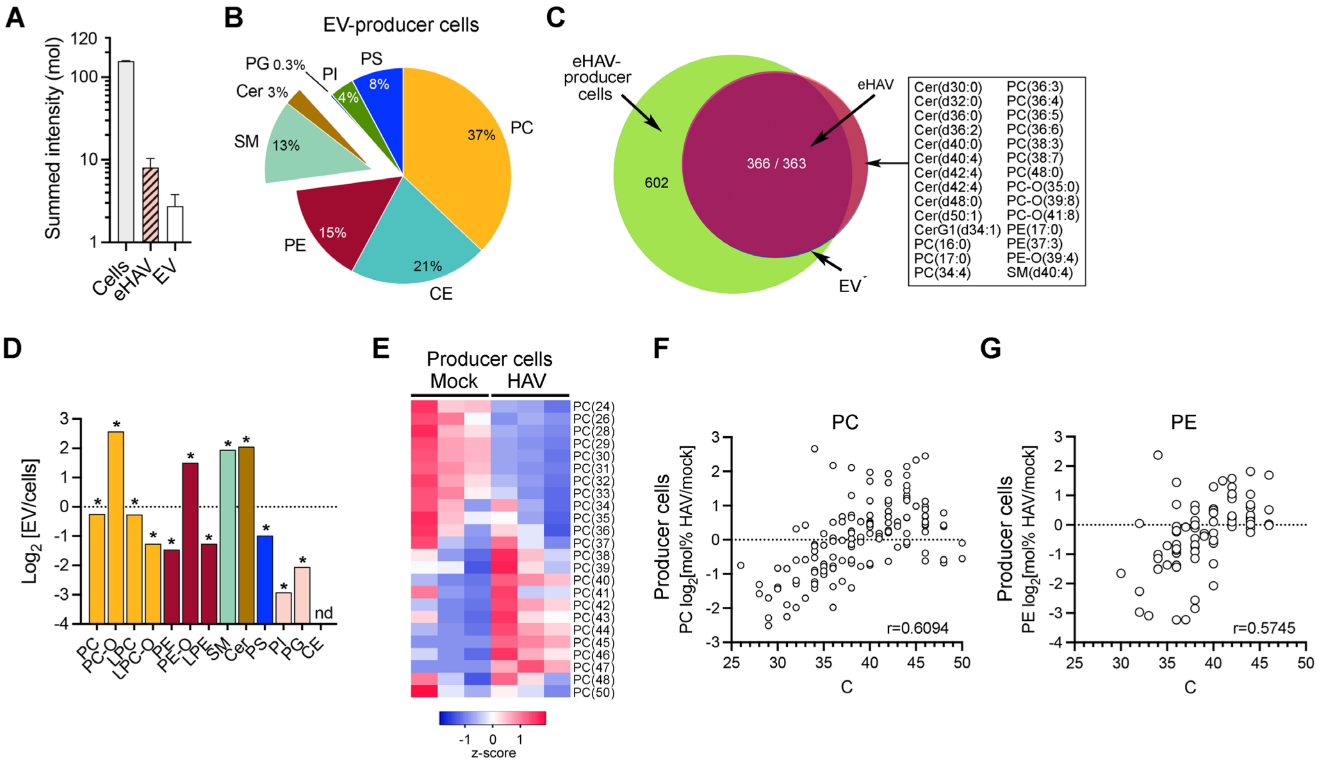

Figure S5. **Lipidomics analysis of eHAV, EV and related producer cells.** (**A)** Summed raw MS intensities of non-glycerolipids in HAV-infected producer cells, eHAV, and control EV samples. (**B)** Normalized percent molar abundance (mol%) of major non-glycerolipid lipids in EV-producer cells. **(C)** Venn diagram of numbers of individual lipid species identified in eHAV (n=366), EV (n=363), and HAV producer cells (n=602). Lipid species listed in the box to the right were identified in eHAV but not in eHAV-producer cells. (**D)** Relative enrichment of major lipid classes in EV versus EV-producer cells shown as $\log_2$ ratios of mol% abundance. *Q<0.01; nd, not detected in EV. (**E)** Heat map showing the relative mol% of individual PC species by total carbon number in HAV-infected eHAV-producer cells versus mock-infected EV-producer cells. **(F)** Correlation between relative mol% abundance of individual PC species in HAV-infected producer cells versus mock-infected control cells versus total carbon number (C). r = Spearman correlation coefficient, $p<0.0001$. **(G)** Similar correlation analysis of PE lipid species. $p<0.0001$.

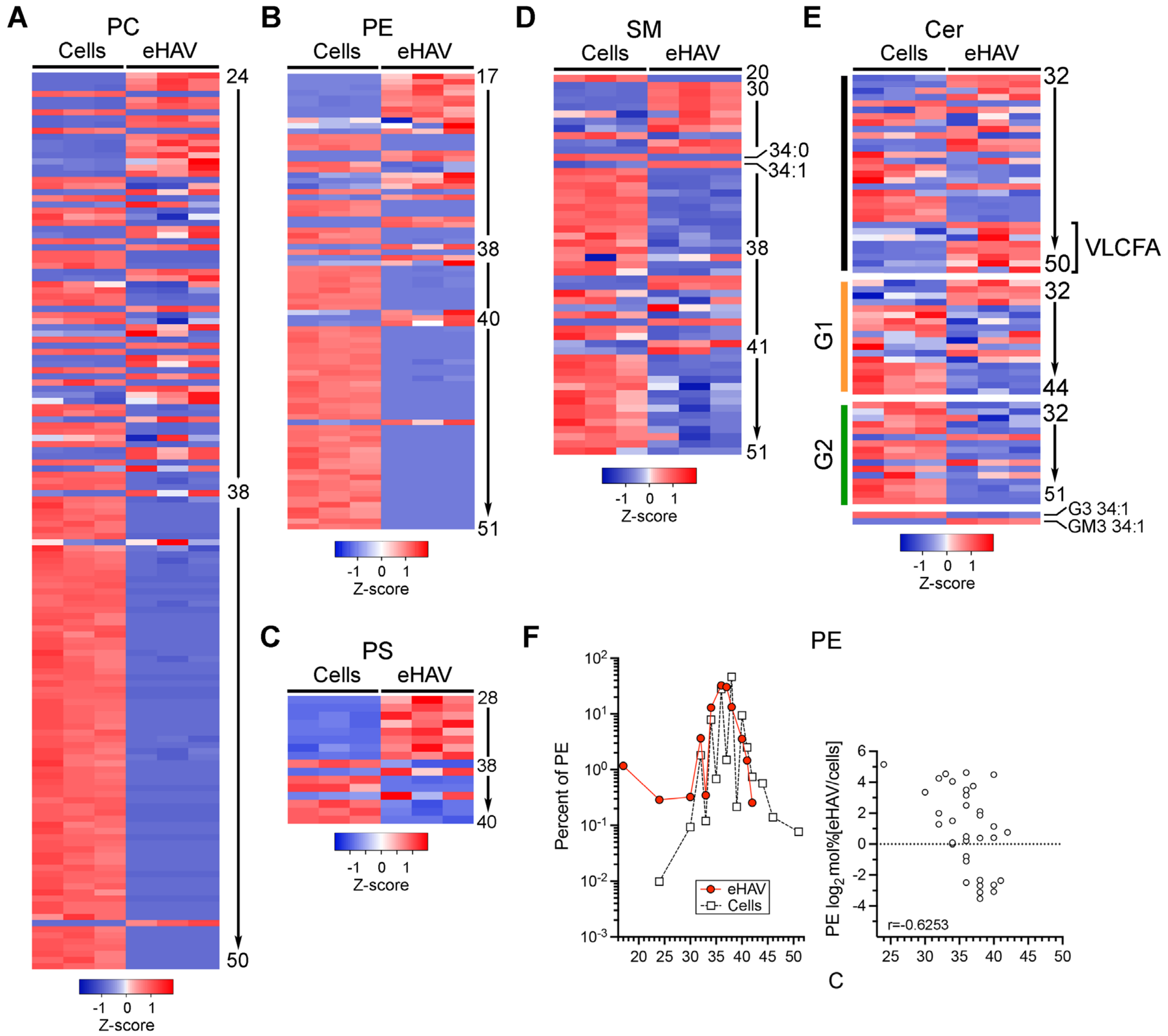

Figure S6. Lipid composition of eHAV versus eHAV-producer cells. (A-E) Heat maps scored on the average molar abundance of individual species as a percent of each major lipid class in eHAV versus cognate producer cells. Lipid species are listed in ascending order by acyl-tail carbon number (shown on the right), and increasing numbers of double bonds. Z-score scales shown below for each heat map. G1, G2, G3 Cer are hexosylceramides. **(F)** (*left*) Normalized mean percent distribution of PE species by carbon number in eHAV and producer cell samples (values shown as percent of PE). (*right*) Correlation between the fold-change in relative molar abundance of individual PE species in eHAV versus producer cells, and total carbon number. r = Spearman correlation coefficient. p=0.0011.

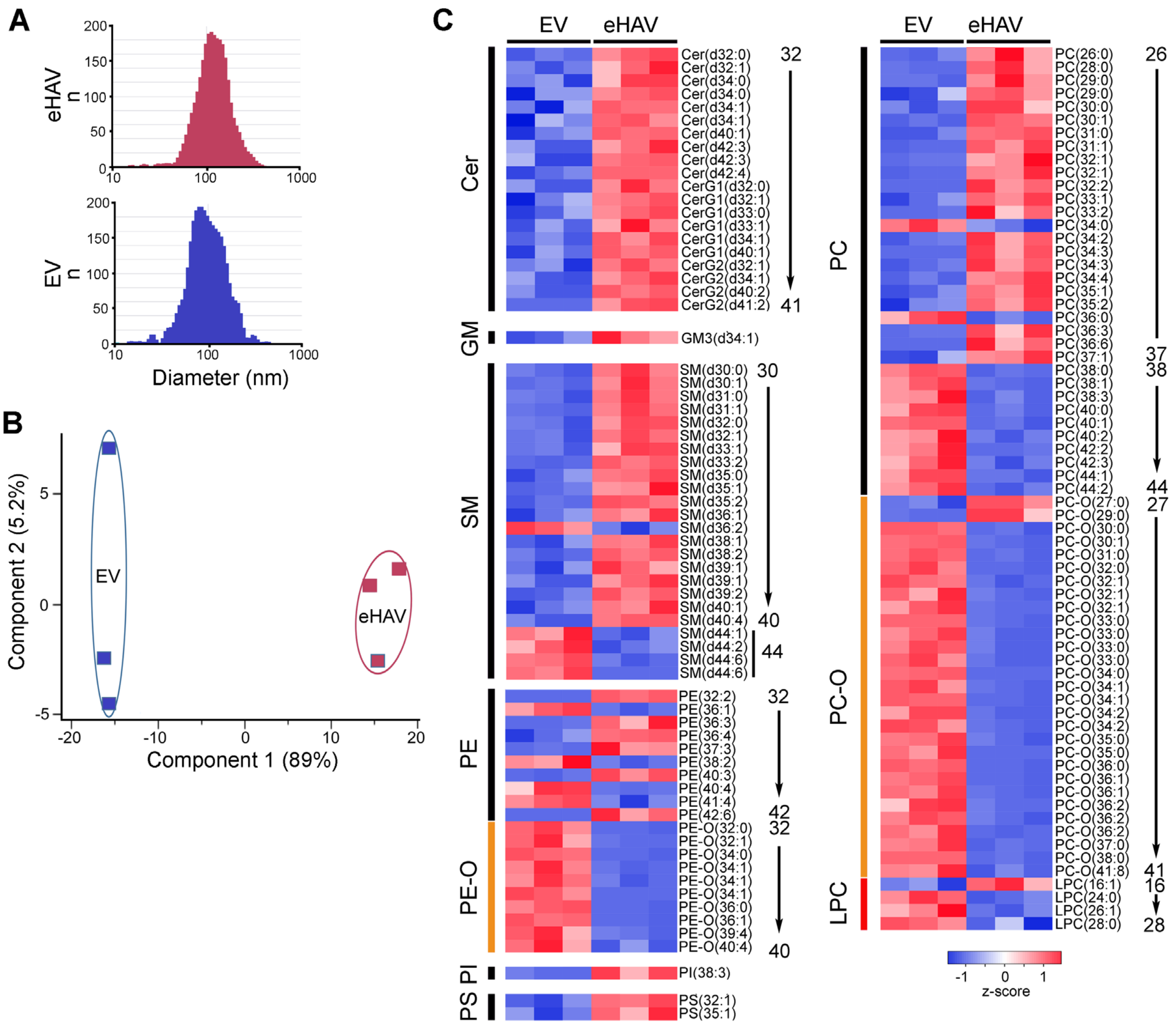

Figure S7. Lipidomics analysis of eHAV versus control EV samples. (**A)** Distribution of particle size determined by ZetaView laser scattering video microscopy analysis of eHAV (*top*, mean diameter 118.3 nm) and EV samples (*bottom*, mean diameter 85.7 nm). **(B)** Principal component analysis of eHAV and EV samples based on normalized intensities of individual commonly-identified lipid species. (**C)** Heat maps showing relative molar abundance of 135 non-glycerolipid species that differed significantly in mol% abundance between eHAV and EV samples (Q<0.01 by two-stage step-up Benjamini-Krieger-Yekutieli method). CerG1 = hexosylceramide, CerG2 = dihexosylceramide.

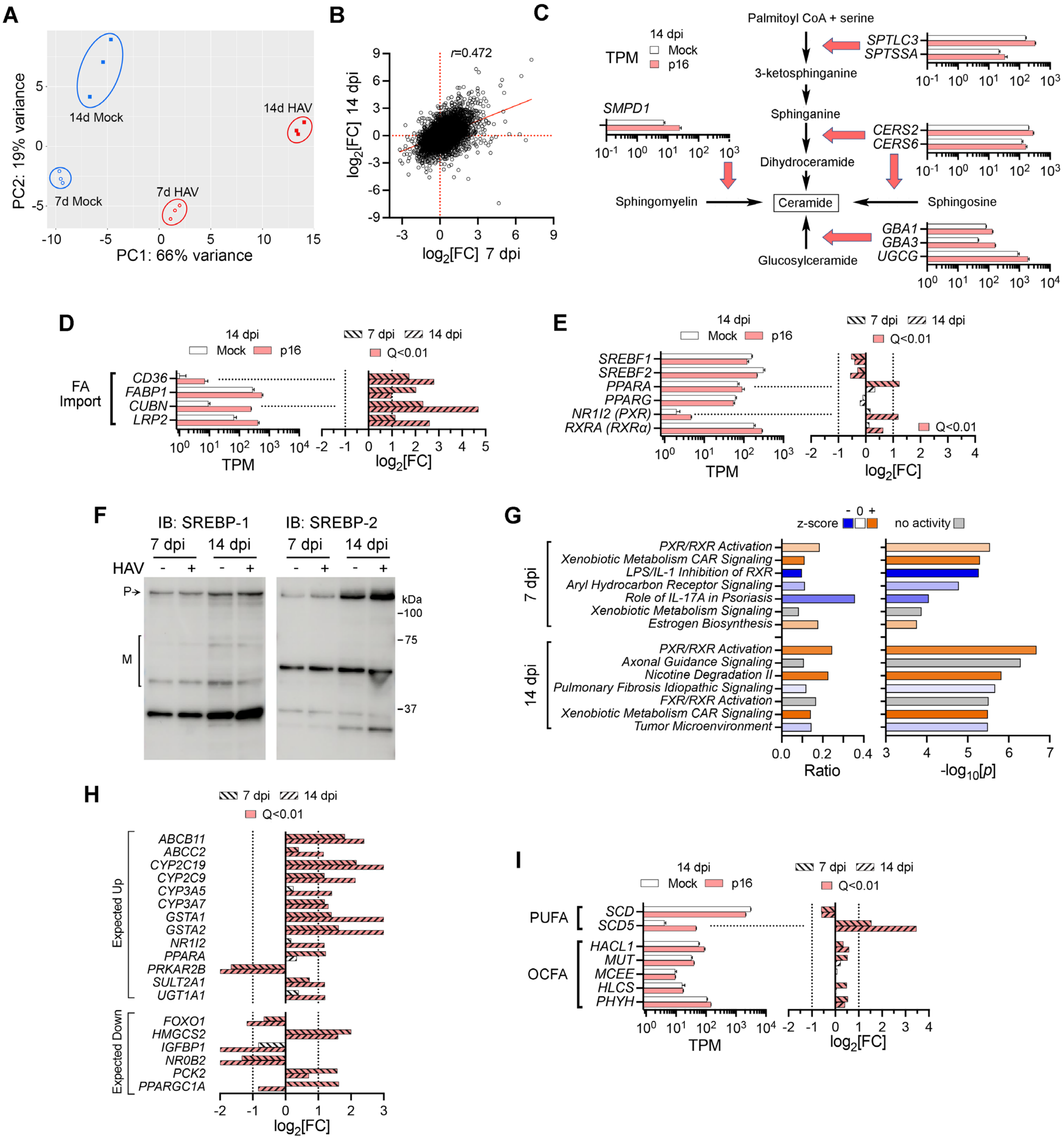

Figure S8. Transcriptomic analysis of HAV-infected Huh-7.5 cells. (A) Principal component analysis of RNAseq data generated by high-throughput sequencing of RNA extracted from mock- or HAV-infected Huh-7.5 cells 7 and 14 days post-infection (dpi). (**B)** Correlation plot of fold-change (FC) in transcript abundance at 7 versus 14 dpi. *r* = spearman correlation coefficient (p<0.0001). (**C)** Transcripts encoding enzymes involved in ceramide synthesis that were increased in infected cells 14 dpi (Q<0.001). Data shown are transcripts per million (TPM) in mock versus

infected cells 14 dpi: SPTLC3, serinepalmitoyltransferase 3; SPTSSA, serinepalmitoyltransferase small subunit A; CERS2, ceramide synthase 2; CERS6, ceramide synthase 6; GBA1, lysosomal acid glucosylceramidase; GBA2, nonlysosomal acid glucosylceramidase; UGCG, ceramide glucosyltransferase; and, SMPD1, acid sphingomyelinase. **(D)** (*left*) Abundance (14 dpi) and (*right*) FC (7 and 14 dpi) of transcripts encoding proteins involved in fatty acid (FA) import. Shaded bars: Q<0.01. (**E)** Abundance (14 dpi) and (*right*) fold-change (7 and 14 dpi) of transcripts of selected lipid-regulatory genes. Shaded bars: Q<0.01. **(F)** Immunoblots of sterol response-element binding protein 1 (SREBP-1) and SREBP-2 in cytosolic lysates of infected Huh-7.5 cells 7 and 14 dpi. P = precursor molecule, M = mature transcription factor. (**G)** Ingenuity Pathway Analysis (IPA) of transcripts differentially regulated >2-fold with Q<0.01 at 7 and 14 dpi. Ratio indicates the proportion of genes associated with each pathway that is differentially regulated by HAV infection. (**H)** HAV-induced changes in transcripts expected to be up-regulated or down-regulated in IPA by PXR/RXR activation at 7 and 14 dpi. **(I)** (*left*) Abundance (14 dpi) and (*right*) fold-change (7 and 14 dpi) of transcripts encoding proteins involved in fatty acid desaturation or generation of odd-chain fatty acids (OCFA).

SUPPLEMENTARY DATASETS

Table S1 Normalized MS intensities of lipid species identified in HAV-infected and mock-infected Huh-7.5 cells.

Table S2 Normalized MS intensities of lipid species identified in HSD17B12-KO54.10 and sgCtrl cells.

Table S3 Normalized MS intensities of lipid species identified in gradient-purified extracellular eHAV and EV.

Table S4 Normalized MS intensities of lipid species identified in eHAV- and EV-producer cells.

Table S5 High-throughput RNA sequencing statistics.

REFERENCES AND NOTES

1. S. M. Lemon, J. J. Ott, P. Van Damme, D. Shouval, Type A viral hepatitis: A summary and update on the molecular virology, epidemiology, pathogenesis and prevention. *J. Hepatol.* **68**, 167–184 (2018).

2. K. H. Jacobsen, Globalization and the changing epidemiology of hepatitis A virus. *Cold Spring Harbor. Perspect. Med.* **8**, a031716 (2018).

3. K. L. McKnight, S. M. Lemon, Hepatitis A virus genome organization and replication strategy. *Cold Spring Harbor. Perspect. Med.* **8**, a033480 (2018).

4. D. B. Smith, P. Simmonds, Classification and genomic diversity of enterically transmitted hepatitis viruses. *Cold Spring Harbor. Perspect. Med.* **8**, a031880 (2018).

5. R. Gosert, D. Egger, K. Bienz, A cytopathic and a cell culture adapted hepatitis A virus strain differ in cell killing but not in intracellular membrane rearrangements. *Virology* **266**, 157–169 (2000).

6. N. L. Teterina, K. Bienz, D. Egger, A. E. Gorbalenya, E. Ehrenfeld, Induction of intracellular membrane rearrangements by HAV proteins 2C and 2BC. *Virology* **237**, 66–77 (1997).

7. Z. Feng, L. Hensley, K. L. McKnight, F. Hu, V. Madden, L. Ping, S.-H. Jeong, C. Walker, R. E. Lanford, S. M. Lemon, A pathogenic picornavirus acquires an envelope by hijacking cellular membranes. *Nature* **496**, 367–371 (2013).

8. A. Das, E. E. Rivera-Serrano, X. Yin, C. M. Walker, Z. Feng, S. M. Lemon, Cell entry and release of quasi-enveloped human hepatitis viruses. *Nat. Rev. Microbiol.* **21**, 573–589 (2023).

9. A. Hirai-Yuki, L. Hensley, J. K. Whitmire, S. M. Lemon, Biliary secretion of quasi-enveloped human hepatitis A virus. *mBio* **7**, e01998–e01916 (2016).

10. K. L. McKnight, L. Xie, O. González-López, X. Chen, S. M. Lemon, Protein composition of the hepatitis A virus quasi-envelope. *Proc. Natl. Acad. Sci. U.S.A.* **114**, 6587–6592 (2017).

11. Y.-H. Chen, W. L. Du, M. C. Hagemeijer, P. M. Takvorian, C. Pau, A. Cali, C. A. Brantner, E. S. Stempinski, P. S. Connelly, H.-C. Ma, P. Jiang, E. Wimmer, G. Altan-Bonnet, N. Altan-Bonnet,

Phosphatidylserine vesicles enable efficient en bloc transmission of enteroviruses. *Cell* **160**, 619–630 (2015).

12. M. Santiana, S. Ghosh, B. A. Ho, V. Rajasekaran, W.-L. Du, Y. Mutsafi, Dennise A De Jésus-Diaz, S. V. Sosnovtsev, E. A. Levenson, G. I. Parra, P. M. Takvorian, A. Cali, C. Bleck, A. N. Vlasova, L. J. Saif, J. T. Patton, P. Lopalco, A. Corcelli, K. Y. Green, N. Altan-Bonnet, Vesicle-cloaked virus clusters are optimal units for inter-organismal viral transmission. *Cell Host Microbe* **24**, 208–220.e8 (2018).

13. J. Morris-Love, G. V. Gee, B. A. O'Hara, B. Assetta, A. L. Atkinson, A. S. Dugan, S. A. Haley, W. J. Atwood, M. Imperiale, D. Galloway, JC polyomavirus uses extracellular vesicles to infect target cells. *MBio* **10**, e00379-19 (2019).

14. S. G. van der Grein, K. A. Y. Defourny, H. H. Rabouw, S. S. Goerdayal, M. J. C. van Herwijnen, R. W. Wubbolts, M. Altelaar, F. J. M. van Kuppeveld, E. N. M. Nolte-'t Hoen, The encephalomyocarditis virus Leader promotes the release of virions inside extracellular vesicles via the induction of secretory autophagy. *Nat. Commun.* **13**, 3625 (2022).

15. X. Han, R. W. Gross, The foundations and development of lipidomics. *J. Lipid Res.* **63**, 100164 (2022).

16. T. Züllig, H. C. Köfeler, High resolution mass spectrometry in lipidomics. *Mass Spectrom. Rev.* **40**, 162–176 (2021).

17. R. W. Jansen, J. E. Newbold, S. M. Lemon, Complete nucleotide sequence of a cell culture-adapted variant of hepatitis A virus: Comparison with wild-type virus with restricted capacity for in vitro replication. *Virology* **163**, 299–307 (1988).

18. A. Das, R. Barrientos, T. Shiota, V. Madigan, I. Misumi, K. L. McKnight, L. Sun, Z. Li, R. M. Meganck, Y. Li, E. Kaluzna, A. Asokan, J. K. Whitmire, M. Kapustina, Q. Zhang, S. M. Lemon, Gangliosides are essential endosomal receptors for quasi-enveloped and naked hepatitis A virus. *Nat. Microbiol.* **5**, 1069–1078 (2020).

19. J. K. Hiltunen, A. J. Kastaniotis, K. J. Autio, G. Jiang, Z. Chen, T. Glumoff, 17B-hydroxysteroid dehydrogenases as acyl thioester metabolizing enzymes. *Mol. Cell. Endocrinol.* **489**, 107–118 (2019).

20. A. Kihara, Very long-chain fatty acids: elongation, physiology and related disorders. *J. Biochem.* **152**, 387–395 (2012).

21. D. Yamane, H. Feng, E. E. Rivera-Serrano, S. R. Selitsky, A. Hirai-Yuki, A. das, K. L. McKnight, I. Misumi, L. Hensley, W. Lovell, O. González-López, R. Suzuki, M. Matsuda, H. Nakanishi, T. Ohto-Nakanishi, T. Hishiki, E. Wauthier, T. Oikawa, K. Morita, L. M. Reid, P. Sethupathy, M. Kohara, J. K. Whitmire, S. M. Lemon, Basal expression of interferon regulatory factor 1 drives intrinsic hepatocyte resistance to multiple RNA viruses. *Nat. Microbiol.* **4**, 1096–1104 (2019).

22. M. Yi, S. M. Lemon, Replication of subgenomic hepatitis A virus RNAs expressing firefly luciferase is enhanced by mutations associated with adaptation of virus to growth in cultured cells. *J. Virol.* **76**, 1171–1180 (2002).

23. B. Mohamed, C. Mazeaud, M. Baril, D. Poirier, A. A. Sow, L. Chatel-Chaix, V. Titorenko, D. Lamarre, Very-long-chain fatty acid metabolic capacity of 17-beta-hydroxysteroid dehydrogenase type 12 (HSD17B12) promotes replication of hepatitis C virus and related flaviviruses. *Sci. Rep.* **10**, 4040 (2020).

24. T. Róg, A. Orłowski, A. Llorente, T. Skotland, T. Sylvänne, D. Kauhanen, K. Ekroos, K. Sandvig, I. Vattulainen, Interdigitation of long-chain sphingomyelin induces coupling of membrane leaflets in a cholesterol dependent manner. *Biochim. Biophys. Acta* **1858**, 281–288 (2016).

25. S. Y. Bu, D. G. Mashek, Hepatic long-chain acyl-CoA synthetase 5 mediates fatty acid channeling between anabolic and catabolic pathways. *J. Lipid Res.* **51**, 3270–3280 (2010).

26. Q. Luo, A. das, F. Oldoni, P. Wu, J. Wang, F. Luo, Z. Fang, Role of ACSL5 in fatty acid metabolism. *Heliyon* **9**, e13316 (2023).

27. R. Kozyraki, J. Fyfe, M. Kristiansen, C. Gerdes, C. Jacobsen, S. Cui, E. I. Christensen, M. Aminoff, A. de la Chapelle, R. Krahe, P. J. Verroust, S. K. Moestrup, The intrinsic factor-vitamin B12 receptor, cubilin, is a high-affinity apolipoprotein A-I receptor facilitating endocytosis of high-density lipoprotein. *Nat. Med.* **5**, 656–661 (1999).

28. J. Ye, R. A. DeBose-Boyd, Regulation of cholesterol and fatty acid synthesis. *Cold Spring Harb. Perspect. Biol.* **3**, a004754 (2011).

29. J. G. Purdy, T. Shenk, J. D. Rabinowitz, Fatty acid elongase 7 catalyzes lipidome remodeling essential for human cytomegalovirus replication. *Cell Rep.* **10**, 1375–1385 (2015).

30. J. Zhou, Y. Zhai, Y. Mu, H. Gong, H. Uppal, D. Toma, S. Ren, R.M. Evans, W. Xie, A novel pregnane X receptor-mediated and sterol regulatory element-binding protein-independent lipogenic pathway. *J. Biol. Chem.* **281**, 15013–15020 (2006).

31. J. Zhang, Y. Wei, B. Hu, M. Huang, W. Xie, Y. Zhai, Activation of human stearoyl-coenzyme A desaturase 1 contributes to the lipogenic effect of PXR in HepG2 cells. *PLOS ONE* **8**, e67959 (2013).

32. M. W. Cho, N. Teterina, D. Egger, K. Bienz, E. Ehrenfeld, Membrane rearrangement and vesicle induction by recombinant poliovirus 2C and 2BC in human cells. *Virology* **202**, 129–145 (1994).

33. D. Paul, S. Hoppe, G. Saher, J. Krijnse-Locker, R. Bartenschlager, Morphological and biochemical characterization of the membranous hepatitis C virus replication compartment. *J. Virol.* **87**, 10612–10627 (2013).

34. K. Iwabuchi, H. Nakayama, C. Iwahara, K. Takamori, Significance of glycosphingolipid fatty acid chain length on membrane microdomain-mediated signal transduction. *FEBS Lett.* **584**, 1642–1652 (2010).

35. Y. Ohno, S. Suto, M. Yamanaka, Y. Mizutani, S. Mitsutake, Y. Igarashi, T. Sassa, A. Kihara, ELOVL1 production of C24 acyl-CoAs is linked to C24 sphingolipid synthesis. *Proc. Natl. Acad. Sci. U.S.A.* **107**, 18439–18444 (2010).

36. M. P. Agbaga, S. Logan, R. S. Brush, R. E. Anderson, Biosynthesis of very long-chain polyunsaturated fatty acids in hepatocytes expressing ELOVL4. *Adv. Exp. Med. Biol.* **801**, 631–636 (2014).

37. E. Koyuncu, J. G. Purdy, J. D. Rabinowitz, T. Shenk, Saturated very long chain fatty acids are required for the production of infectious human cytomegalovirus progeny. *PLOS Pathog.* **9**, e1003333 (2013).

38. P. Fraisl, H. Tanaka, S. Forss-Petter, H. Lassmann, Y. Nishimune, J. Berger, A novel mammalian bubblegum-related acyl-CoA synthetase restricted to testes and possibly involved in spermatogenesis. *Arch. Biochem. Biophys.* **451**, 23–33 (2006).

39. L. Chen, R. P. Vasoya, N. H. Toke, A. Parthasarathy, S. Luo, E. Chiles, J. Flores, N. Gao, E. M. Bonder, X. Su, M. P. Verzi, HNF4 regulates fatty acid oxidation and is required for renewal of intestinal stem cells in mice. *Gastroenterology* **158**, 985–999.e9 (2020).

40. D. G. Mashek, M. A. McKenzie, C. G. Van Horn, R. A. Coleman, Rat long chain acyl-CoA synthetase 5 increases fatty acid uptake and partitioning to cellular triacylglycerol in McArdle-RH7777 cells. *J. Biol. Chem.* **281**, 945–950 (2006).

41. J. A. Nchoutmboube, E. G. Viktorova, A. J. Scott, L. A. Ford, Z. Pei, P. A. Watkins, R. K. Ernst, G. A. Belov, Increased long chain acyl-Coa synthetase activity and fatty acid import is linked to membrane synthesis for development of picornavirus replication organelles. *PLOS Pathog.* **9**, e1003401 (2013).

42. K. Trajkovic, C. Hsu, S. Chiantia, L. Rajendran, D. Wenzel, F. Wieland, P. Schwille, B. Brügger, M. Simons, Ceramide triggers budding of exosome vesicles into multivesicular endosomes. *Science* **319**, 1244–1247 (2008).

43. K. Yuyama, H. Sun, D. Mikami, T. Mioka, K. Mukai, Y. Igarashi, Lysosomal-associated transmembrane protein 4B regulates ceramide-induced exosome release. *FASEB J.* **34**, 16022–16033 (2020).

44. T. Skotland, K. Sagini, K. Sandvig, A. Llorente, An emerging focus on lipids in extracellular vesicles. *Adv. Drug Deliv. Rev.* **159**, 308–321 (2020).

45. T. Skotland, N. P. Hessvik, K. Sandvig, A. Llorente, Exosomal lipid composition and the role of ether lipids and phosphoinositides in exosome biology. *J. Lipid Res.* **60**, 9–18 (2019).

46. E. E. Rivera-Serrano, O. Gonzalez-Lopez, A. Das, S. M. Lemon, Cellular entry and uncoating of naked and quasi-enveloped human hepatoviruses. *eLife* **8**, e43983 (2019).

47. E. Bonsergent, E. Grisard, J. Buchrieser, O. Schwartz, C. Théry, G. Lavieu, Quantitative characterization of extracellular vesicle uptake and content delivery within mammalian cells. *Nat. Commun.* **12**, 1864 (2021).

48. M. I. Morandi, P. Busko, E. Ozer-Partuk, S. Khan, G. Zarfati, Y. Elbaz-Alon, P. Abou Karam, T. Napso Shogan, L. Ginini, Z. Gil, N. Regev-Rudzki, O. Avinoam, Extracellular vesicle fusion visualized by cryo-electron microscopy. *PNAS Nexus* **1**, pgac156 (2022).

49. S. M. Lemon, P. C. Murphy, P. A. Shields, L. H. Ping, S. M. Feinstone, T. Cromeans, R. W. Jansen, Antigenic and genetic variation in cytopathic hepatitis A virus variants arising during persistent infection: evidence for genetic recombination. *J. Virol.* **65**, 2056–2065 (1991).

50. A. Das, A. Hirai-Yuki, O. González-López, B. Rhein, S. Moller-Tank, R. Brouillette, L. Hensley, I. Misumi, W. Lovell, J. M. Cullen, J. K. Whitmire, W. Maury, S. M. Lemon, TIM1 (HAVCR1) Is not essential for cellular entry of either quasi-enveloped or naked hepatitis A virions. *MBio* **8**, e00969-17 (2017).

51. Y. Li, I. Misumi, T. Shiota, L. Sun, E. M. Lenarcic, H. Kim, T. Shirasaki, A. Hertel-Wulff, T. Tibbs, J. E. Mitchell, K. L. McKnight, C. E. Cameron, N. J. Moorman, D. R. McGivern, J. M. Cullen, J. K. Whitmire, S. M. Lemon, The ZCCHC14/TENT4 complex is required for hepatitis A virus RNA synthesis. *Proc. Natl. Acad. Sci. U.S.A.* **119**, e2204511119 (2022).

52. H. Kim, D. Aponte-Diaz, M. S. Sotoudegan, D. Shengjuler, J. J. Arnold, C.E. Cameron, The enterovirus genome can be translated in an IRES-independent manner that requires the initiation factors eIF2A/eIF2D. *PLOS Biol.* **21**, e3001693 (2023).

53. A. J. Pruijssers, A. S. George, A. Schäfer, S. R. Leist, L. E. Gralinksi, K. H. Dinnon III, B. L. Yount, M. L. Agostini, L. J. Stevens, J. D. Chappell, X. Lu, T. M. Hughes, K. Gully, D. R. Martinez, A. J. Brown, R. L. Graham, J. K. Perry, V. du Pont, J. Pitts, B. Ma, D. Babusis, E. Murakami, J. Y. Feng, J. P. Bilello, D. P. Porter, T. Cihlar, R. S. Baric, M. R. Denison, T. P. Sheahan, Remdesivir inhibits SARS-CoV-2 in human lung cells and chimeric SARS-CoV expressing the SARS-CoV-2 RNA polymerase in mice. *Cell Rep.* **32**, 107940 (2020).

54. D. Yamane, D. R. McGivern, E. Wauthier, M.K. Yi, V. J. Madden, C. Welsch, I. Antes, Y. Wen, P. E. Chugh, C. E. McGee, D. G. Widman, I. Misumi, S. Bandyopadhyay, S. Kim, T. Shimakami, T. Oikawa, J. K. Whitmire, M. T. Heise, D. P. Dittmer, C. C. Kao, S. M. Pitson, A. H. Merrill Jr., L. M. Reid, S. M. Lemon, Regulation of the hepatitis C virus RNA replicase by endogenous lipid peroxidation. *Nat. Med.* **20**, 927–935 (2014).

55. K. J. Blight, J. A. McKeating, C. M. Rice, Highly permissive cell lines for subgenomic and genomic hepatitis C virus RNA replication. *J. Virol.* **76**, 13001–13014 (2002).

56. A. MacGregor, M. Kornitschuk, J. G. Hurrell, N. I. Lehmann, A. G. Coulepis, S. A. Locarnini, I. D. Gust, Monoclonal antibodies against hepatitis A virus. *J. Clin. Microbiol.* **18**, 1237–1243 (1983).

57. R. M. Pellegrino, A. Di Veroli, A. Valeri, L. Goracci, G. Cruciani, LC/MS lipid profiling from human serum: A new method for global lipid extraction. *Anal. Bioanal. Chem.* **406**, 7937–7948 (2014).

58. N. Vu, M. Narvaez-Rivas, G. Y. Chen, M. J. Rewers, Q. Zhang, Accurate mass and retention time library of serum lipids for type 1 diabetes research. *Anal. Bioanal. Chem.* **411**, 5937–5949 (2019).

59. Z. Li, Q. Zhang, Ganglioside isomer analysis using ion polarity switching liquid chromatography-tandem mass spectrometry. *Anal. Bioanal. Chem.* **413**, 3269–3279 (2021).

60. B. MacLean, D. M. Tomazela, N. Shulman, M. Chambers, G. L. Finney, B. Frewen, R. Kern, D. L. Tabb, D. C. Liebler, M. J. MacCoss, Skyline: an open source document editor for creating and analyzing targeted proteomics experiments. *Bioinformatics* **26**, 966–968 (2010).

61. Y. Benjamini, A. Krieger, D. Yekutieli, Adaptive linear step-up procedures that control the false discovery rate. *Biometrika* **93**, 491–507 (2006).

62. S. Tyanova, J. Cox, Perseus: A bioinformatics platform for integrative analysis of proteomics data in cancer research. *Methods Mol. Biol.* **1711**, 133–148 (2018).

63. M. Narvaez-Rivas, N. Vu, G. Y. Chen, Q. Zhang, Off-line mixed-mode liquid chromatography coupled with reversed phase high performance liquid chromatography-high resolution mass spectrometry to improve coverage in lipidomics analysis. *Anal. Chim. Acta* **954**, 140–150 (2017).

64. M. Narváez-Rivas, Q. Zhang, Comprehensive untargeted lipidomic analysis using core-shell C30 particle column and high field orbitrap mass spectrometer. *J. Chromatogr. A* **1440**, 123–134 (2016).

65. S. Babicki, D. Arndt, A. Marcu, Y. Liang, J. R. Grant, A. Maciejewski, D. S. Wishart, Heatmapper: Web-enabled heat mapping for all. *Nucleic Acids Res.* **44**, W147–W153 (2016).
